# Clonal Analyses Reveal the Impact of Hematopoietic Stem and Progenitor Cell Aging on T Cell Development

**DOI:** 10.1111/acel.70615

**Published:** 2026-06-29

**Authors:** Julia Gensheimer, Jessica LaGosh, Emma R. Moulton, Victoria Sun, Stephanie C. de Barros, Encarnacion Montecino‐Rodriguez, Gloria Yiu, Xuegang Yuan, Kenneth Dorshkind, Gay M. Crooks

**Affiliations:** ^1^ Molecular Biology Interdepartmental Program University of California, Los Angeles Los Angeles California USA; ^2^ Department of Pathology and Laboratory Medicine, David Geffen School of Medicine University of California, Los Angeles Los Angeles California USA; ^3^ Division of Rheumatology, Department of Medicine, David Geffen School of Medicine University of California, Los Angeles Los Angeles California USA; ^4^ Molecular Biology Institute University of California, Los Angeles Los Angeles California USA; ^5^ Broad Stem Cell Research Center, David Geffen School of Medicine University of California, Los Angeles Los Angeles California USA; ^6^ Jonsson Comprehensive Cancer Center, David Geffen School of Medicine University of California, Los Angeles Los Angeles California USA; ^7^ Department of Pediatrics, David Geffen School of Medicine University of California, Los Angeles Los Angeles California USA

**Keywords:** aging, bone marrow, hematopoiesis, hematopoietic stem cells, thymus gland, T‐lymphocytes

## Abstract

T cell output from the thymus falls throughout life and is associated with profound remodeling of the thymic stroma. To what extent the decline in T cell output is caused by aging of the hematopoietic stem and progenitor cells (HSPCs) has been difficult to define because of HSPC heterogeneity, the multi‐stage process of HSPC migration, and the cross‐talk between hematopoietic and stromal elements of the thymus. To address the contribution of HSPC aging on T cell development, we interrogated T cell differentiation of phenotypically defined HSPCs from young and aged bone marrow using the Artificial Thymic Organoid (ATO) system, an in vitro model which allows quantification of T cell differentiation from single HSPCs within a controlled microenvironment. Phenotypically, most HSCs from young bone marrow were CD150^lo^ lymphoid‐biased (Ly‐HSC), whereas aged HSCs were predominantly CD150^hi^ myeloid‐biased (My‐HSC). Clonal analysis showed Ly‐HSCs had greater T cell potential than My‐HSCs, but aging had little if any impact on T cell output from the same immunophenotypic HSC. Further, clonal studies of early thymic progenitors (ETPs) demonstrated comparable T cell potential in young and aged cells. We conclude that the hematopoietic contribution to thymic insufficiency during aging is likely due to a relative shift in the aged bone marrow to myeloid‐biased HSCs, rather than a per cell loss of T cell potential across HSPC in general. The profound changes that occur in the thymic microenvironment during aging likely also provide a major contribution to defects in thymopoiesis.

## Graphical abstract

This study explored whether the decline in thymic output with age is due to intrinsic defects in aged hematopoietic stem and progenitor cells. With age, the proportion of lymphoid‐biased (Ly‐)HSCs decreased in the bone marrow and early thymic progenitor (ETP) cell number declined in the thymus. However when comparing single aged HSCs and ETPs in the artificial thymic organoid (ATO), T cell potential was higher in Ly‐HSC than My‐HSC but was similar for each subtype irrespective of age.

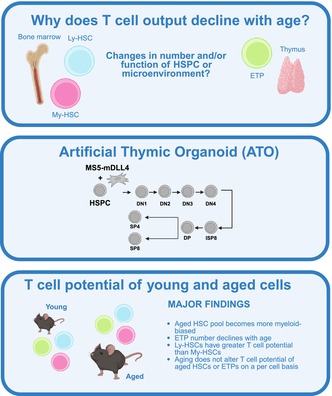

## 1 Introduction

Immune function declines with age, leading to reduced vaccination efficacy and increased susceptibility to infections and cancers in older adults (Goyani et al. 2024; Montecino‐Rodriguez et al. 2013). Although the hematopoietic stem cells (HSCs) of the bone marrow are the origin of all hematopoietic components of the immune system, commitment of HSCs and progenitors to the T cell lineage is initiated only after migration from the bone marrow through the circulation and seeding of the thymus, where the microenvironment provides complex intercellular and molecular signals for T cell development (Rothenberg et al. 2008). Progressive loss of thymic microenvironment integrity begins early in life, and aging is associated with a dramatic decline in T cell output (Montecino‐Rodriguez et al. 2013). The dual anatomic sites involved in hematopoiesis and T cell differentiation, and the additional complexity involved in cross‐talk between hematopoietic and stromal cells in the thymus, make it challenging to assess the specific contribution of the hematopoietic (non‐stromal) compartment to reduced T cell production with aging (Rothenberg et al. 2008).

Murine bone marrow transplantation assays have provided much of the functional information on aging of HSCs and progenitors (HSPCs). Some studies have shown that aged bone marrow HSPCs have intrinsic defects of bone marrow engraftment and decreased B lymphoid potential (Kim et al. 2003; Liang et al. 2005; Miller and Allman 2003; Morrison et al. 1996; Rossi et al. 2005; Tyan 1977). The role of HSC aging on T cell development remains elusive due to limited approaches to directly quantitate T cell potential (Ogden and Mickliem 1976; Ross et al. 1982). In transplantation assays, HSCs must first home, engraft and proliferate in the bone marrow, and then migrate to the thymus to recapitulate normal steady state thymopoiesis, resulting in egress of mature naïve T cells into the circulation (Rothenberg et al. 2008). Thus, as the appearance of donor‐derived T cells in peripheral blood occurs weeks after initial engraftment, readout of T cell potential of HSCs in these studies is very indirect (Rossi et al. 2005). Furthermore, the use of pre‐transplant irradiation can affect both the recipient bone marrow and thymic microenvironments (Dorshkind et al. 2020; Xiao et al. 2017).

Several reports have addressed these challenges with in vitro culture systems (Aspinall and Andrew 2001; Eren et al. 1988; Zediak et al. 2007) such as fetal thymic organ cultures (FTOC) (Nitta et al. 2013) and the OP9‐DL1 monolayer culture system (Schmitt and Zuniga‐Pflucker 2002). However, conclusions from these reports have been inconsistent; while some studies report a reduction in T cell output from aged HSPCs (Eren et al. 1988; Zediak et al. 2007), others show no difference in T cell potential with age (Aspinall and Andrew 2001). The discrepancies between the results of in vitro studies and the difficulties in quantifying T cell potential from specific HSPCs in vivo emphasize the need for systems to evaluate T cell potential in a controlled microenvironment.

Investigation of intrinsic defects in aged HSCs is further complicated by HSC heterogeneity (Dorshkind et al. 2020). Through serial repopulation assays of clonally derived HSCs, myeloid‐ (My‐) and lymphoid‐ (Ly‐) biased HSCs were found to retain stable lineage bias even after secondary transplants (Muller‐Sieburg et al. 2004, 2002). My‐ and Ly‐HSCs were later defined by relative expression of CD150 after studies showed that CD150^hi^ HSCs had increased myeloid cell production compared to CD150^lo^ HSCs (Beerman et al. 2010; Challen et al. 2010). The aged HSC pool is associated with a greater frequency and number of CD150^hi^ My‐HSCs compared to CD150^lo^ Ly‐HSCs (Beerman et al. 2010; Challen et al. 2010; Cho et al. 2008). This myeloid‐skewing of HSCs likely accounts for the preponderance of mature myeloid cells and increased inflammatory cytokines in aged bone marrow as well as increased rates of myeloproliferative disease with aging (Dorshkind et al. 2020; Rollison et al. 2008). It should be noted that populations defined as Ly‐HSC and My‐HSC possess a relative bias in lineage potential and both Ly‐ and My‐HSC populations retain some potential for the alternative lineage (Montecino‐Rodriguez et al. 2019; Muller‐Sieburg et al. 2004). Clonal studies of single well‐defined HSPCs offer the ability to examine the functional heterogeneity that exists within each phenotypic population that may be missed in cultures from bulk populations.

Early thymic progenitors (ETPs) represent the most immature identifiable pre‐T cell commitment population in the thymus (Allman et al. 2003). ETPs decline in number as early as 3 months of age in mice (Srinivasan et al. 2023), a finding thought to be due to age‐associated defects in the thymic stromal niches that result in decreased Notch signaling. However, whether the intrinsic T cell potential of ETPs changes with age remains unclear. Using FTOCs, we previously reported that aging reduces the T lymphoid potential of ETPs (Min et al. 2004); however, we seeded bulk ETPs into fetal thymic lobes, so the developmental potential of individual ETPs is unknown.

The in vitro Artificial Thymic Organoid (ATO) system allows for detailed dissection and quantification of T cell potential from single precursors isolated from the thymic and bone marrow microenvironments (Montel‐Hagen et al. 2019, 2020; Seet et al. 2017). The mouse ATO system efficiently recapitulates thymopoiesis through positive selection from single HSPCs, with kinetics that reflect the stage in the hematopoietic hierarchy of the initiating cell (Montel‐Hagen et al. 2020). In the current studies, data from clonal analysis in the ATO system suggest that the phenotypically defined lineage bias of each HSC determines its T cell potential irrespective of age. Our findings suggest that aging of the hematopoietic compartment has a role in causing reduced T cell output primarily, but this is limited to the low proportion of Ly‐HSC in the aged bone marrow compared to young bone marrow rather than a global loss of lymphoid potential in aged HSCs.

## 2 Results

### 2.1 T Cell Output and Differentiation From Bone Marrow Hematopoietic Stem and Progenitor Cells (HSPCs) During Aging

**FIGURE 1:**
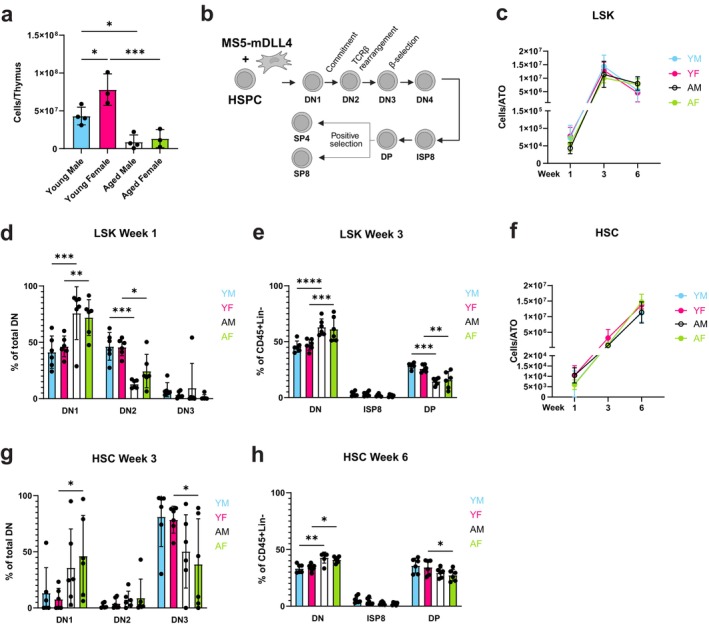
Bulk young and aged hematopoietic stem and progenitor cells (HSPCs) produce similar cell output in vitro but young HSPCs develop more rapidly. (a) Mean cell numbers from thymi harvested from young (7–8 weeks old) and aged (18–24 months old) male and female C57BL/6J mice. Each dot represents an individual experiment with average of pooled data from 2 to 6 mice. Error bar denotes ± SD (*n* = 54 mice, ordinary one‐way ANOVA). (b) Schematic of the mouse ATO system and key stages of T cell development. (c) Mean cell numbers at weeks 1, 3, and 6 of ATO initiated from bulk LSKs (Lineage‐Sca1+kit+) from young (7–8 weeks old) and aged (18.5–24 months old) male and female C57BL/6J mice. Each dot represents an average of pooled data from 12 ATOs. Error bar denotes ± SD (*n* = 144 ATOs total from two independent experiments, ordinary two‐way ANOVA). (d) Frequencies of subsets of double negative (DN) cells (TCRβ‐CD3‐CD4‐CD8‐) at week 1 of ATO initiated from bulk LSKs isolated from young and aged male and female C57BL/6J mice, shown as a percentage of total DN cells. Each dot represents two pooled ATOs. Error bar denotes ± SD (*n* = 48 ATOs total from two independent experiments, ordinary two‐way ANOVA). (e) Frequencies of cell subsets at week 3 of ATO initiated from bulk LSKs from young and aged male and female C57BL/6J mice. Frequencies of DN cells, immature single‐positive CD8+ (ISP8) cells (TCRβ‐CD3‐CD8+CD4‐), and double‐positive (DP) cells (TCRβ‐CD3‐CD8+CD4+) are shown as a percentage of total live CD45+Lin‐ cells. Each dot represents two pooled ATOs. Error bar denotes ± SD (*n* = 48 ATOs total from two independent experiments, ordinary two‐way ANOVA). (f) Mean cell numbers at weeks 1, 3, and 6 of ATO initiated from bulk HSCs (LSK CD150+CD48‐) from young (7–8 weeks old) and aged (18.5–24 months old) male and female C57BL/6J mice. Each dot represents an average of pooled data from 12 ATOs. Error bar denotes ± SD (*n* = 144 ATOs total from 2 independent experiments, ordinary two‐way ANOVA). (g) Frequencies of subsets of DN cells at week 3 of ATO initiated from bulk HSCs from young and aged male and female C57BL/6J mice, shown as a percentage of total DN cells. Each dot represents two pooled ATOs. Error bar denotes ± SD (*n* = 48 ATOs total from two independent experiments, ordinary two‐way ANOVA). (h) Frequencies of cell subsets at week 6 of ATO initiated from bulk HSCs from young and aged male and female C57BL/6J mice. Frequencies of DN cells, ISP8 cells, and DP cells are shown as a percentage of total live CD45+Lin‐ cells. Each dot represents two pooled ATOs. Error bar denotes ± SD (*n* = 48 ATOs total from two independent experiments, ordinary two‐way ANOVA). AF, aged female; AM, aged male; YF, young female; YM, young male. For all statistical analyses, only significant values are shown. A *p*‐value of < 0.05 was deemed significant (*p ≤ 0.05, **p ≤ 0.01, ***p ≤ 0.001, ****p ≤ 0.0001).

Consistent with the well documented loss of thymic cellularity during aging (Montecino‐Rodriguez et al. 2013), both male and female C57BL/6J mice showed a significant decrease in thymocyte numbers with age (Figure 1a). To explore if the reason for lower thymocyte cell numbers in aged mice was due to a reduction in intrinsic T cell potential of the bone marrow HSPCs that seed the thymus, we used the ATO model, an in vitro system that employs a controlled artificial thymic microenvironment to induce T commitment and differentiation of different types of HSPCs (Montel‐Hagen et al. 2020; Seet et al. 2017) (Figure 1b). Each bulk ATO was initiated with 50–100 Lineage‐Sca1+kit+ (LSK) bone marrow cells (a population which includes both HSC and progenitor cells (Spangrude et al. 1988)), isolated by fluorescence‐activated cell sorting (FACS) from young (7 to 8 weeks old) or aged (18–24 months old) mice (Figure S1a,b), and aggregated with the murine stromal cell line MS5 engineered to express the Notch ligand murine Delta‐like ligand 4 (MS5‐mDLL4) (Montel‐Hagen et al. 2020).

T cell development was assessed at Weeks 1, 3, and 6 in the ATO system via cell counting and flow cytometry (Figure S1c). In normal thymopoiesis, distinct stages of differentiation are defined by cell surface phenotypes, beginning at the CD4‐CD8‐ double negative (DN) stage which comprises DN1 through DN4 stages based on CD44 and CD25 expression. After the DN stages, TCRβ‐CD3‐ murine thymocytes acquire CD8 expression to become immature single‐positive 8 (ISP8) cells, then upregulate CD4 expression to become CD4+CD8+ double positive (DP) cells, and then undergo positive selection to produce either single positive CD4+CD8‐ (SP4) or CD4‐CD8+ (SP8) cells.

LSK bone marrow cells produced similar numbers of total cells at Weeks 1, 3, and 6 of ATO culture irrespective of age and sex (Figure 1c), but differentiation was more rapid from young LSKs; by Week 1, young LSKs had differentiated to the DN2 stage whereas aged LSKs remained largely at DN1 (Figure 1d, Figure S2a). At Week 3 with further differentiation, cultures from young LSKs contained significantly more DP cells than those from aged LSKs, which remained largely at the DN stage (Figure 1e, Figure S2b). A similar trend favoring T cell differentiation from young LSKs was seen at Week 6 but was not statistically significant (Figure S2c,d).

We next focused our studies on the rare CD150+CD48‐ subset of LSKs which is highly enriched for long‐term repopulating HSCs (Kiel et al. 2005). Each bulk ATO was initiated with 50–100 HSCs and again, young and aged HSCs produced similar total cell output at all‐time points (Figure 1f). As expected, differentiation from less mature HSCs was delayed relative to differentiation from LSK with 100% of all cultures remaining at the DN1 stage at Week 1 (Figure S2e,f). By Week 3 however, cultures from young and aged HSC revealed different phenotypes with more progeny from young HSC cultures progressing to the DN3 stage and cultures from aged HSCs retaining more immature (DN1) cells (Figure 1g, Figure S2g). At Week 6, DP cells were detectable in all cultures but were more frequent in those from young HSCs (Figure 1h, Figure S2h). Together, our data demonstrate that although total cell yield from bulk cultures of young and aged HSCs is similar, progression through T cell differentiation is more rapid from young HSCs than aged HSCs in vitro.

### 2.2 Aged HSCs Produce More Myeloid Cells in Vitro and Contain a Greater Proportion of Myeloid‐Biased HSCs

**FIGURE 2:**
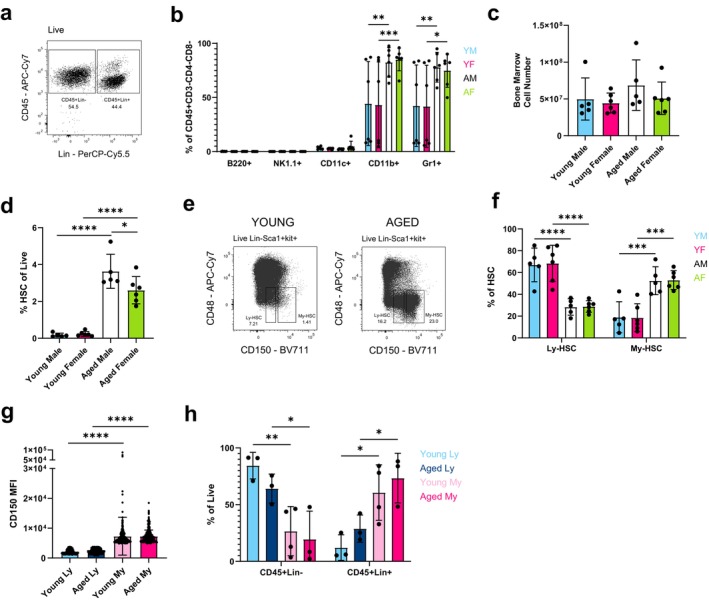
Aged HSCs produce more myeloid cells in vitro and have a greater proportion of My‐HSCs. (a) Representative FACS plot of CD45+Lin+ and CD45+Lin‐ cell populations in a single ATO, shown as a percentage of total live cells. Lin includes B220, NK1.1, Ter119, CD11c, CD11b, and Gr1 markers. (b) Frequencies of B, NK, and myeloid cells at week 3 of ATO initiated from bulk HSCs from young and aged male and female C57BL/6J mice, shown as a percentage of total live CD45+CD3‐CD4‐CD8‐ cells. Each dot represents two pooled ATOs. Error bar denotes ± SD (*n* = 48 ATOs total from 2 independent experiments, ordinary two‐way ANOVA). (c) Mean cell numbers from bone marrow harvested from young (7 to 8 weeks old) and aged (18–24 months old) and male and female C57BL/6J mice. Each dot represents an individual experiment with average of pooled data from 2 to 6 mice. Error bar denotes ± SD (*n* = 79 mice, ordinary one‐way ANOVA). (d) Frequency of HSCs from young and aged male and female C57BL/6J Lin‐depleted mouse bone marrow, shown as a percentage of total live Lin‐depleted cells. Each dot represents an individual experiment with average of pooled data from 2 to 6 mice. Error bar denotes ± SD (*n* = 79 mice, ordinary one‐way ANOVA). (e) Representative FACS plots of young and aged bone marrow. My‐HSCs (CD150^hi^CD48) and Ly‐HSCs (CD150^lo^CD48‐) are shown as a percentage of LSK (gated from live LSK cells). Data are concatenated from 59 mice from six independent experiments into one FACS plot. (f) Frequencies of Ly‐ and My‐HSCs from young and aged male and female C57BL/6J mouse bone marrow, shown as a percentage of HSCs. Each dot represents an individual experiment with average of pooled data from 2 to 6 mice. Error bar denotes ± SD (*n* = 79 mice, ordinary two‐way ANOVA). (g) CD150 BV711 mean fluorescence intensity (MFI) of single Ly‐ and My‐HSCs from young and aged mice obtained via index sorting. MFI was not significantly different between young and aged HSCs within their respective HSC phenotypes. Each dot represents one cell. Error bar denotes ± SD (*n* = total 1734 single cells total from 59 mice from six independent experiments, ordinary one‐way ANOVA). (h) Frequencies of CD45+Lin‐ and CD45+Lin+cells at week 4 of ATO initiated from bulk Ly‐ and My‐HSCs from young and aged C57BL/6J mice, shown as a percentage of total live cells. Each dot represents one ATO. Error bar denotes ± SD (*n* = 13 ATOs total from four independent experiments, ordinary two‐way ANOVA). AF, aged female; AM, aged male; YF, young female; YM, young male. For all statistical analyses, only significant values are shown. A *p*‐value of < 0.05 was deemed significant (*p ≤ 0.05, **p ≤ 0.01, ***p ≤ 0.001, ****p ≤ 0.0001).

While assessing T cell differentiation from young and aged HSPCs, we observed significant production of other lineages in some of the ATO cultures. These experiments were initially performed using a non‐T lineage (Lin+) antibody cocktail labeled with a common fluorophore in the flow cytometry panel, comprised of B, NK, and myeloid cell markers (Lin: B220, NK1.1, Ter119, CD11c, CD11b, and Gr1) (Figure 2a). To determine the specific cell types in the Lin+ gate, we assessed expression of individual B, NK, and myeloid cell markers. The dominant non‐T lineage cells were of myeloid origin (CD11b+and/or Gr1+), and aged HSCs produced higher frequencies and numbers of myeloid cells in the ATOs compared to HSCs from young mice (Figure 2b, Figure S3a).

We hypothesized that the above findings may be due to previously reported differences between the myeloid‐biased subset of HSCs (My‐HSCs) and lymphoid‐biased HSCs (Ly‐HSCs) in the bone marrow of young and aged mice (Beerman et al. 2010; Cho et al. 2008). Although total bone marrow cell numbers of hindlimb bones were not different between young and aged mice (Figure 2c), aging had a marked effect on the composition of HSPCs and HSCs in the bone marrow; both frequency and number of LSKs and CD150+CD48‐ HSCs were significantly higher in aged mice than young mice, irrespective of sex (Figure 2d, Figure S3b–e).

In addition, the HSC pool was dominated by CD150^hi^ My‐HSC in aged bone marrow, whereas in young bone marrow, CD150^lo^ Ly‐HSC were significantly more frequent (Figure 2e,f, Figure S3f). Nonetheless, the total number of both Ly‐ and My‐HSCs was significantly greater in aged than in young bone marrow (Figure S3g) because of the significantly higher number of total HSCs in aged bone marrow (Figure S3b) (Montecino‐Rodriguez et al. 2019).

To determine if aging affects the functional lineage potential of phenotypic Ly‐ and My‐HSCs, we isolated each population from young and aged bone marrow based on CD150 surface expression levels (Figure 2g) and seeded 25–50 of each cell type into ATOs. Bulk cultures of My‐HSCs produced a greater frequency of Lin+cells (determined to be myeloid cells) compared to Ly‐HSCs regardless of age (Figure 2h). These data with bulk cultures prompted us to study single phenotypically defined young and aged Ly‐ and My‐HSCs to determine T cell potential of these HSC subsets at a clonal level.

### 2.3 T Cell Potential is Higher From Single Ly‐HSC Than my‐HSC Regardless of Age

**FIGURE 3:**
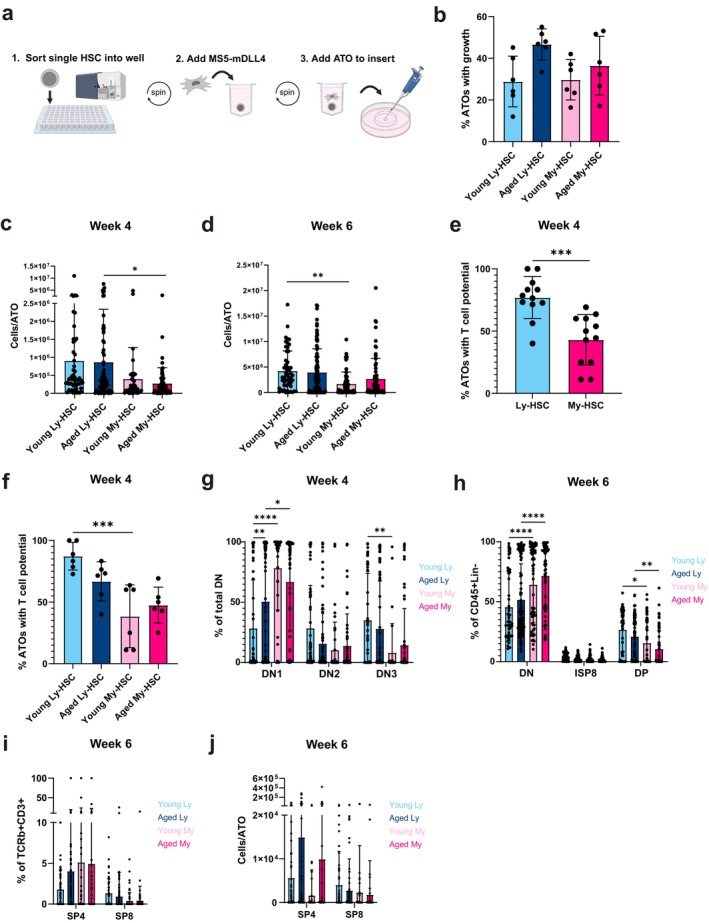
Single young and aged Ly‐HSCs demonstrate greater T cell potential and cell output compared to My‐HSCs regardless of age. (a) Schematic of the mouse single cell ATO system. The well represents each well of a V‐bottom 96 well plate. (b) Cloning efficiency (percentage of ATOs with growth) from single young (8 weeks old) and aged (18–24 months old) Ly‐ and My‐HSC ATOs. Each dot represents an individual experiment. Error bar denotes ± SD (*n* = 1441 ATOs total from six independent experiments, ordinary one‐way ANOVA). (c) Cell numbers at week 4 of ATO initiated from single Ly‐ or My‐HSC from young and aged C57BL/6J mice. Each dot represents one ATO. Error bar denotes ± SD (*n* = 234 ATOs total from six independent experiments, ordinary one‐way ANOVA). (d) Cell numbers at week 6 of ATO initiated from single Ly‐ or My‐HSC from young and aged C57BL/6J mice. Each dot represents one ATO. Error bar denotes ± SD (*n* = 275 ATOs total from six independent experiments, ordinary one‐way ANOVA). (e) T cell potential at week 4 of ATO initiated from single Ly‐ or My‐HSC, shown as a percentage of ATOs with growth. Age groups are shown together. T cell potential is defined as expression of TCRβ, CD3, CD4, CD8, and/or presence of DN2 (CD44+CD25+) or DN3 (CD44‐CD25+) cells. Each dot represents an individual experiment. Error bar denotes ± SD (*n* = 234 ATOs total from six independent experiments, Welch's *t*‐test). (f) T cell potential at Week 4 of ATO initiated from single Ly‐ or My‐HSC from young and aged C57BL/6J mice, shown as a percentage of ATOs with growth. Age groups are shown separately. Each dot represents an individual experiment. Error bar denotes ± SD (*n* = 234 ATOs total from six independent experiments, ordinary one‐way ANOVA). (g) Frequencies of subsets of DN cells at week 4 of ATO initiated from single Ly‐ or My‐HSC from young and aged C57BL/6J mice, shown as a percentage of total DN cells. Each dot represents one ATO. Error bar denotes ± SD (*n* = 234 ATOs total from six independent experiments, ordinary two‐way ANOVA). (h) Frequencies of cell subsets at week 6 of ATO initiated from single Ly‐ or My‐HSC from young and aged C57BL/6J mice, shown as a percentage of total live CD45+Lin‐ cells. Each dot represents one ATO. Error bar denotes ± SD (*n* = 275 ATOs total from six independent experiments, ordinary two‐way ANOVA). (i) Frequencies of SP4 and SP8 cells at week 6 of ATO initiated from single Ly‐ or My‐HSC from young and aged C57BL/6J mice, shown as a percentage of TCRβ+CD3+ cells. Each dot represents one ATO. Error bar denotes ± SD (*n* = 275 ATOs total from 6 independent experiments, ordinary two‐way ANOVA). (j) Cell numbers of SP4 and SP8 cells at week 6 of ATO initiated from single Ly‐ or My‐HSC from young and aged C57BL/6J mice, calculated using total cell numbers and frequency of live cells. Each dot represents one ATO. Error bar denotes ± SD (*n* = 275 ATOs total from 6 independent experiments, ordinary two‐way ANOVA). For all statistical analyses, only significant values are shown. A *p*‐value of < 0.05 was deemed significant (*p ≤ 0.05, **p ≤ 0.01, ***p ≤ 0.001, ****p ≤ 0.0001).

Single Ly‐ or My‐HSC from the bone marrow of young and aged mice were isolated by FACS into each well of 96 well plates and combined with MS5‐mDLL4 cells to create single cell ATOs with modifications from the existing protocol (Montel‐Hagen et al. 2020) (Figure 3a). Single cell ATOs were first classified via flow cytometry as having “growth” or “no growth” based on the presence or absence respectively of cells in the “FSC v SSC”, live, and CD45+ gates (Figure S4a,b). Mean cloning efficiency (i.e., percentage of ATOs with growth) ranged from 28% to 46% depending on the starting population (Figure 3b). Ly‐HSCs produced significantly higher total cell numbers than My‐HSCs regardless of age but cell output from each clone was not significantly different between young and aged Ly‐HSCs or from young and aged My‐HSCs (Figure 3c,d). Thus, HSC phenotype determined clonal output rather than HSC age.

We next assessed the T cell potential of single Ly‐ and My‐HSCs. T cell potential was defined as expression by flow cytometry of any one or more of the following T cell markers: TCRβ, CD3, CD4, and/or CD8 and/or the presence of DN2 or DN3 cells (Figure S4b,c). Of the clones with growth, 77% (± 16%) exhibited T cell potential if seeded from Ly‐HSCs compared to 43% (±20%) from My‐HSCs (*p* = 0.0002) (Figure 3e). This finding prevailed regardless of age, as young and aged Ly‐HSCs both exhibited higher T cell potential than age‐matched My‐HSCs (Figure 3f). There was a trend of increased T cell potential of Ly‐HSC from young compared to aged, but this did not reach statistical significance (Figure 3f).

Close evaluation of early T cell differentiation revealed significant differences between single Ly‐ and My‐HSCs. At Week 4, DN cells dominated all clones (Figure S5a), but the number of DN cells in individual clones from Ly‐HSC was significantly higher than My‐HSCs regardless of age (Figure S5b). In addition, within the DN population, Ly‐HSCs (both young and aged) differentiated more rapidly, producing a higher frequency and number of cells that had reached the DN3 stage, compared to My‐HSCs, which generated clones most of which remained DN1 (Figure 3g, Figure S5c). The same relative pattern of differentiation was seen at Week 6; cultures from both young and aged Ly‐HSCs contained more cells that had advanced to the DP stage than cultures from My‐HSCs; the latter cultures contained significantly higher proportions of immature DN cells (Figure 3h, Figure S5d). Our observation that single Ly‐HSCs differentiated more efficiently than single My‐HSCs in clonal analyses likely explains the difference seen in bulk cultures of unfractionated HSCs from young and aged bone marrow (Figure 1d,e,g,h), the latter containing a higher proportion of My‐HSCs.

Analysis of mature SP4 and SP8 cells in Week 6 cultures showed a wide variation in frequency and cell number but no significant difference between young and aged Ly‐ and My‐HSCs, suggesting that once T cell commitment was established in a clone, further differentiation was not affected by the lineage bias or age of the initiating HSC (Figure 3i,j). This is consistent with the finding that nearly all clones with growth contained T lineage cells by week 6 (Figure S5e). Cultures also produced a wide range of frequencies and numbers of all TCRβ+CD3+ and TCRβ+CD3+DP (termed “DP late”) cells with no significant difference in cell output (Figure S5f–j).

### 2.4 Categorization of Clones Based on Lineage Differentiation

**FIGURE 4:**
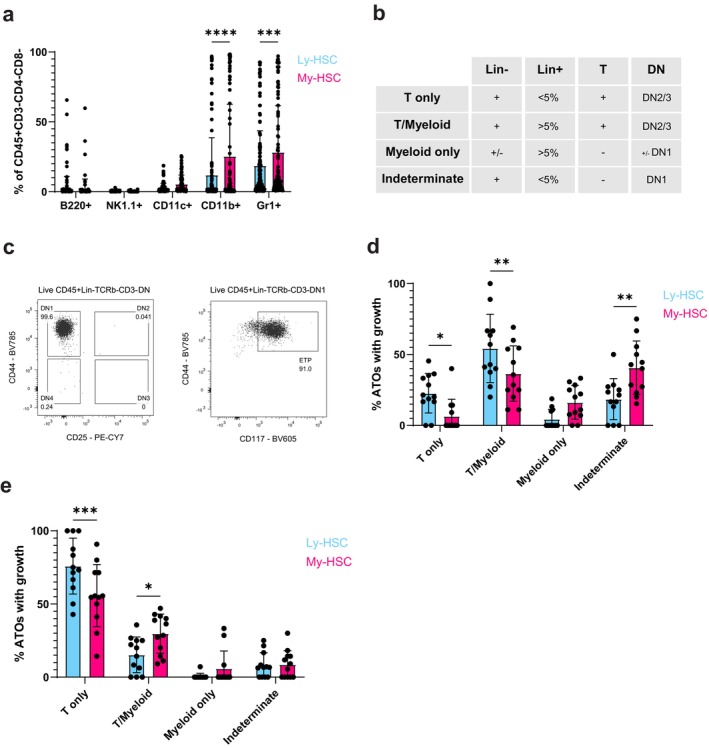
Single My‐HSCs produce more myeloid and indeterminate lineage phenotypes compared to Ly‐HSCs. (a) Frequencies of B, NK, and myeloid cells at weeks 4 and 6 of ATO initiated from single Ly‐ or My‐HSC, shown as a percentage of total live CD45+CD3‐CD4‐CD8‐ cells. Age groups are shown together. Each dot represents one ATO. Error bar denotes ± SD (*n* = 262 ATOs total from six independent experiments, ordinary two‐way ANOVA). (b) Definition of ATO lineage phenotypes from single cell Ly‐ and My‐HSC from young and aged C57BL/6J mice: T only, T/myeloid, myeloid only, and indeterminate. (c) Representative FACS plot of ATO with DN and early thymic progenitor (ETP) cell production, seen in myeloid only and indeterminate ATO lineage phenotypes. Frequencies of DN1‐4 are shown as a percentage of total DN cells; frequency of ETP is shown as a percentage of DN1 cells. (d) Frequencies of ATO lineage phenotypes at week 4 of ATO initiated from single Ly‐ or My‐HSC, shown as a percentage of ATOs with growth. Age groups are shown together. Each dot represents an individual experiment. Error bar denotes ± SD (*n* = 234 ATOs total from six independent experiments, ordinary two‐way ANOVA). (e) Frequencies of ATO lineage phenotypes at week 6 of ATO initiated from single Ly‐ or My‐HSC, shown as a percentage of ATOs with growth. Age groups are shown together. Each dot represents an individual experiment. Error bar denotes ± SD (*n* = 275 ATOs total from six independent experiments, ordinary two‐way ANOVA). For all statistical analyses, only significant values are shown. A *p*‐value of < 0.05 was deemed significant (*p ≤ 0.05, **p ≤ 0.01, ***p ≤ 0.001, ****p ≤ 0.0001).

Similar to bulk cultures, myeloid and T cell differentiation could be detected in certain clones. Clones from My‐HSCs produced a greater frequency of CD11b+and Gr1+ cells compared to Ly‐HSCs (Figure 4a) and these trends were present regardless of age (Figure S6a).

To summarize the lineages produced in all the clones from single Ly‐ and My‐HSCs, we defined lineage output in each ATO as one of four categories: T only, T/myeloid, myeloid only, and indeterminate (Figure 4b). T only clones had the definite presence of T lineage cells and minimal if any myeloid cell production (Figure S6b). T/myeloid clones were defined as clearly having both T and myeloid cells (Figure S6c). Myeloid only clones had > 5% Lin+(predominantly myeloid) cells with no signs of T lineage cells; all DN cells in myeloid only clones were phenotypically DN1 (Figure S6d). Indeterminate clones had 100% of cells at the DN1 stage, oftentimes with a phenotype similar to early thymic progenitors (ETPs) (Figure 4c, Figure S6e).

At week 4, almost all Ly‐HSC clones identified as either T only or T/myeloid, with a significantly higher percentage of ATOs in these two categories compared to My‐HSC ATOs (Figure 4d). In contrast, My‐HSC ATOs had a significantly higher percentage of ATOs at the indeterminate stage and a trend toward more myeloid only clones compared to Ly‐HSC ATOs. By week 6, Ly‐HSC ATOs still had a significantly greater percentage of T only clones compared to My‐HSC ATOs, which had significantly more clones classified as T/myeloid (Figure 4e). These data further confirmed the myeloid bias intrinsic to My‐HSCs compared to Ly‐HSCs, irrespective of age (Figure S6f–g).

### 2.5 Early Thymic Progenitors (ETPs) From Young and Aged Mice Exhibit Similar T Cell Output and Differentiation Kinetics in Vitro

**FIGURE 5:**
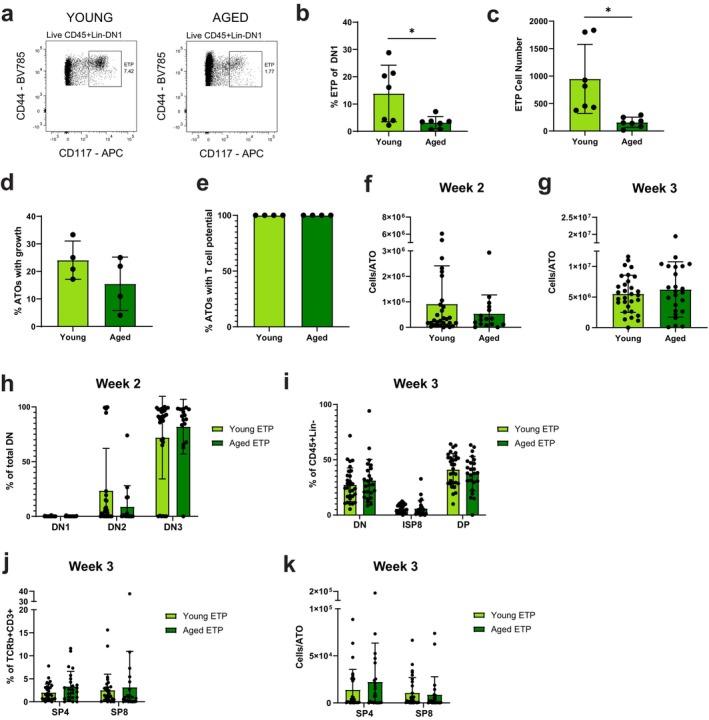
T cell potential of single aged early thymic progenitors (ETPs) are similar to young in vitro. (a) Representative FACS plots of young and aged thymus. ETPs are shown as a percentage of DN1 cells (gated from live Lin‐ DN cells). Data are concatenated from 54 mice from six independent experiments into one FACS plot. (b) Frequency of ETPs from young and aged C57BL/6J mouse thymus, shown as a percentage of DN1 cells. Each dot represents an individual experiment with average of pooled data from 2 to 6 mice. Error bar denotes ± SD (*n* = 54 mice, Welch's *t*‐test). (c) Cell numbers of ETPs from young and aged C57BL/6J mouse thymus, calculated from total cell numbers and ETP frequency as a percentage of live cells. Each dot represents an individual experiment with average of pooled data from 2 to 6 mice. Error bar denotes ± SD (*n* = 54 mice, Welch's *t*‐test). (d) Cloning efficiency (percentage of ATOs with growth) from single young (7–8 weeks old) and aged (21–24 months old) ETP ATOs. Each dot represents an individual experiment. Error bar denotes ± SD (*n* = 624 ATOs total from four independent experiments, Welch's *t*‐test). (e) T cell potential at weeks 2, 3, and 6 of ATO initiated from single ETP from young and aged C57BL/6J mice, shown as a percentage of ATOs with growth. Each dot represents an individual experiment. Error bar denotes ± SD (*n* = 130 ATOs total from four independent experiments, Welch's *t*‐test). (f) Cell numbers at week 2 of ATO initiated from single ETP from young and aged C57BL/6J mice. Each dot represents 1 ATO. Error bar denotes ± SD (*n* = 46 ATOs total from four independent experiments, Welch's *t*‐test). (g) Cell numbers at week 3 of ATO initiated from single ETP from young and aged C57BL/6J mice. Each dot represents one ATO. Error bar denotes ± SD (*n* = 54 ATOs total from 4 independent experiments, Welch's *t*‐test). (h) Frequencies of subsets of DN cells at week 2 of ATO initiated from single ETP from young and aged C57BL/6J mice, shown as a percentage of total DN cells. Each dot represents one ATO. Error bar denotes ± SD (*n* = 46 ATOs total from four independent experiments, multiple unpaired *t*‐test). (i) Frequencies of cell subsets at Week 3 of ATO initiated from single ETP from young and aged C57BL/6J mice. Frequencies of DN cells, ISP8 cells, and DP cells are shown as a percentage of total live CD45+Lin‐ cells. Each dot represents 1 ATO. Error bar denotes ± SD (*n* = 54 ATOs total from four independent experiments, multiple unpaired *t*‐test). (j) Frequencies of SP4 and SP8 cells at Week 3 of ATO initiated from single ETP from young and aged C57BL/6J mice, shown as a percentage of TCRβ+CD3+ cells. Each dot represents one ATO. Error bar denotes ± SD (*n* = 54 ATOs total from four independent experiments, multiple unpaired *t*‐test). (k) Cell numbers of SP4 and SP8 cells at Week 3 of ATO initiated from single ETP from young and aged C57BL/6J mice, calculated using total cell numbers and frequency of live cells. Each dot represents one ATO. Error bar denotes ± SD (*n* = 54 total ATOs from four independent experiments, multiple unpaired *t*‐test). For all statistical analyses, only significant values are shown. A *p*‐value of < 0.05 was deemed significant (*p ≤ 0.05, **p ≤ 0.01, ***p ≤ 0.001, ****p ≤ 0.0001).

We next examined the impact of aging on ETPs, the most immature progenitors resident in the thymus. The frequency of ETPs (defined as the kit^hi^ subset of DN1 cells) (Figure 5a) within the DN1 population was significantly lower in aged thymi (Figure 5b), although this difference was driven entirely by data from female mice (Figure S7a). Nonetheless, because of the drop in total thymocyte numbers with aging (Figure 1a), the total ETP cell number per thymus was significantly lower in aged compared to young mice of both sexes (Figure 5c).

We next tested if the dramatic decline in thymus cellularity with age could be secondary to an intrinsic loss of T cell potential in aged ETPs by analyzing growth in ATOs from single ETPs isolated from young and aged thymi (Figure S7b,c). Mean cloning efficiency ranged from 15% to 24%, with a non‐significant trend of young ETPs having higher cloning efficiency than aged ETPs (Figure 5d). Regardless of thymus age, all clones from ETPs were identified as T only clones as ATOs had no evidence of myeloid differentiation (Figure 5e). At all‐time points analyzed, differentiation and cell output from ETPs was similar whether ATOs were initiated from young or aged mice (Figure 5f–i, Figure S8a–i). Young and aged ETPs also produced similar frequencies and numbers of mature TCRβ+CD3+ cells including DP late, SP4, and SP8 (Figure 5j,k, Figure S9a–e). These data suggest that although ETP numbers are significantly lower in the aged thymi, their capacity to generate T cells on a per cell basis in vitro is similar to young ETPs.

We previously reported increased apoptosis and decreased proliferation of aged ETPs when comparing them with young adolescent (4–6 weeks old) thymocytes (Min et al. 2004); however, in our current study, when comparing young adult (10–13 week old) with aged (20–21 months old) mice, we did not detect any significant differences in apoptosis or cell cycling; this suggests that intrinsic ETP function changes little after the early adolescent period (Figure S10a–e).

## 3 Discussion

The question of whether HSPCs inherently lose T cell potential with age or if the decline in T cell output seen in vivo is secondary to changes in the aged bone marrow or thymic microenvironment is highly relevant for the rational development of therapeutics that target immune aging. The HSC pool changes dramatically with age, increasing both in number and myeloid‐biased differentiation (Cho et al. 2008; Dykstra et al. 2011). Aged HSPCs have been reported to have decreased self‐renewal and impaired homing and engraftment (Liang et al. 2005; Morrison et al. 1996; Rossi et al. 2005). However, most studies supporting this conclusion have used heterogeneous populations of HSPCs and are often confounded by irradiation prior to transplantation, making it difficult to discern the intrinsic T cell potential of aged HSPCs in steady state conditions (Dorshkind et al. 2020).

A recent study addressed the shortcomings of prior transplantation studies by using antibody mediated HSC elimination to condition recipient animals; with this approach, they found transplantation of young HSCs into aged hosts supported “youthful” hematopoietic characteristics, notably a higher contribution of naïve T cells from young donor HSCs than aged host‐derived cells (Konturek‐Ciesla et al. 2025). However, transplantation of bulk HSCs from young mice would include a higher proportion of Ly‐HSCs compared to My‐HSCs, which confounds a conclusion of an intrinsic advantage of individual young HSCs. To our knowledge, all previous in vitro studies of T cell potential have also examined the output from bulk populations of HSPCs, obscuring the ability to quantitate the lineage potential of individual cells.

We previously found young and aged Ly‐HSCs transplanted into young mice conditioned with busulfan rather than irradiation showed no defects in B lymphopoiesis (Montecino‐Rodriguez et al. 2019). When T cell potential of single young and aged Ly‐HSCs was assessed in the current study, aging did not intrinsically alter the T cell potential of individual Ly‐HSCs. Together, these data suggest that aged Ly‐HSCs retain normal lymphoid potential. While we did see a non‐significant trend of reduced T cell potential from aged Ly‐HSCs compared to young Ly‐HSCs, and we acknowledge some intrinsic defects in aged HSPCs cannot be ruled out from our studies, it seems unlikely that the impact of these subtle differences would be sufficient to produce the profound drop in T cell numbers in the endogenous thymus.

Our studies support the conclusion that changes in composition of the bone marrow HSC pool during aging, rather than an overall loss of T cell potential in each HSC, at least partially determines the loss of T cell production. Young and aged My‐HSCs have similar defects in T cell potential, augmenting early transplantation studies that showed equivalent repopulation ability of the bone marrow between young and aged My‐HSCs (Cho et al. 2008). In a recent report, depletion of My‐HSCs using an anti‐CD150 antibody enhanced immune response to infection after vaccination (J. B. Ross et al. 2024), suggesting that the accumulation of My‐HSCs with age contributes to a dysfunctional immune system. My‐HSC depletion decreased peripheral effector memory T cells, suggesting a reduction in peripheral inflammation; however, no clear effect on thymic function was shown.

Myeloid‐skewing of the aged HSC pool is thought to be caused in part by increased inflammatory cytokine production during aging (Montecino‐Rodriguez et al. 2019; Thomas et al. 2020). This chronic state of inflammation, termed inflammaging, increases inflammatory signals that drive normal HSC proliferation and differentiation in some settings while impairing self‐renewal in others (Fotopoulou et al. 2025; Pietras 2017). Further, the rise in cellular senescence, increased myeloid cell production, and enhanced adipogenesis in the aged bone marrow may initiate the production of proinflammatory cytokines that induce myelopoiesis and inhibit lymphopoiesis (Aguilar‐Navarro et al. 2020; Fotopoulou et al. 2025; Pietras 2017). While our studies do not address the question directly, the production of myeloid cells and subsequently inflammatory cytokines from My‐HSCs could have contributed to the delay in T cell differentiation in the ATO. Alternatively, the DN1 phenotype produced by many My‐HSC clones is non‐specific and could identify generation of other non‐T cells rather than delayed T cell developmental kinetics.

A strength of our study, namely the uniform in vitro ATO microenvironment, might also be considered a potential limitation as it removes the impact of proinflammatory changes in the aged thymic microenvironment such as the age‐related loss of thymic epithelial cells and the gain of adipocytes which may amplify subtle intrinsic defects that only manifest under inflammatory conditions in vivo (Kousa et al. 2024; Montecino‐Rodriguez et al. 2013). This prompted us to study ETPs exposed to the aged thymic microenvironment in the ATO. We previously reported intrinsic defects in T lymphoid potential of aged ETPs in our studies using FTOCs (Min et al. 2004); however, in the longer term ATO we showed equivalent T cell potential in young and aged ETPs. This discrepancy may relate to the different in vitro conditions of the two assays and the slower differentiation kinetics of the ATO that could potentially afford aged ETPs the opportunity to reprogram any intrinsic defects.

Our previous studies reported increased apoptosis and decreased proliferation in aged ETPs when comparing to ETPs from young adolescent (4–6 weeks old) mice (Min et al. 2004). However, our current study showed no difference in apoptosis or proliferation between young adult (10–13 weeks old) and aged (20–21 months old) mice. Another report showed increased apoptosis in cultures of aged DN cells, specifically DN2 and DN3 cells (Andrew and Aspinall 2001); however, our studies found no difference in apoptosis or proliferation between freshly isolated thymocyte DN populations. Further studies are needed to define patterns of apoptosis and proliferation of thymocytes across all developmental stages.

It remains unclear why the number of ETPs in the thymus declines with age; however, we report that the clonal capacity of an ETP for T cell differentiation was at most mildly affected by age and unlikely to be the sole cause of the massive decrease in thymus output with age. Our findings are supportive of studies suggesting that alterations within the thymic microenvironment are a primary cause of reduced cellularity and output of the organ with age (Kousa et al. 2024; Srinivasan et al. 2023). However, we acknowledge that our studies did not measure these factors directly and further studies are needed to elucidate the mechanism for ETP loss in the aged thymus, including possible alterations in homing or proliferation of upstream progenitors. It is not known whether aged HSCs have an altered ability to home and engraft the thymus, nor whether Ly‐HSCs have a greater propensity to home to the thymus compared to My‐HSCs.

One potential limitation of our study is the artificial nature of the ATO with high levels of Notch ligand expression and IL‐7, factors which are not only essential for T cell development but also thought to aid in rejuvenation of aged cells (Andrew and Aspinall 2001; Montecino‐Rodriguez et al. 2013). It is possible that restorative factors in the ATO system could theoretically rescue the T cell potential of aged HSPCs. However, we have previously shown using a transgenic Notch reporter mouse model that Notch signaling followed the same pattern and intensity during T cell differentiation in the endogenous thymus and in the ATOs generated from bone marrow LSK cells (Montel‐Hagen et al. 2020).

To our knowledge, our work is the first to directly study T cell differentiation and output from single Ly‐ and My‐HSCs and thymic progenitors, permitting a quantitative assessment of the effects of aging on individual cells within these phenotypically defined populations. Further, our approach eliminates the influence of the aged thymic microenvironment and allows for the interrogation of T cell potential in a constant setting. Nonetheless, the aging of the bone marrow microenvironment likely has a role in skewing the HSC pool toward a myeloid bias. Our data suggest that therapies that target both the bone marrow and thymic microenvironments could be most effective for restoration of T cell output during aging.

## 4 Materials and Methods

### 4.1 Cell Lines

The MS5‐mDLL4 cell lines were generated in our laboratory as previously described (Montel‐Hagen et al. 2020). Stable expression was confirmed by flow cytometry for DLL4 expression after several weeks of culture.

### 4.2 Mice

All animal experiments were conducted under a protocol approved by the UCLA Chancellor's Animal Research Committee. This study used young (7–8 weeks old) and aged (18–24 months old) male and female C57BL/6J (Cat #JAX:000664) mice from the Jackson Laboratory (Bar Harbor, Maine) and the National Institute on Aging (Bethesda, MD). For cell cycling and apoptosis assays, young mice were 10–13 weeks old and aged mice were 20–21 months old.

### 4.3 Bone Marrow Harvest and Preparation

Mice were euthanized by high concentrations of isoflurane (Butler Schein, Dublin, OH) followed by cervical dislocation. Both femur, tibia, and fibula bones (six bones per mouse) were dissected and kept intact in RPMI 1640 (CellGro, Cat #10‐040‐CV) at 4°C for no more than 16 h overnight as previously described (Papazian et al. 2017). Typically, bones were crushed for bulk ATO experiments and flushed for single cell ATO experiments. Bones were crushed with a mortar and pestle while suspended in MACS buffer (PBS/0.5% bovine serum albumin/2 mM EDTA). For flushing, the ends of individual leg bones were cut and flushed with a 3 mL syringe and 25G needle with MACS buffer. The cell suspensions were added to a 50 μm nylon cell strainer (Falcon), rinsed with MACs buffer, and kept on ice.

Cells were centrifuged at 400 g for 5 min at 4°C. The supernatant was aspirated and red blood cells were lysed with 3 mL of 1× ACK lysis buffer (Gibco/ThermoFisher Scientific) at room temperature for 3 min, after which 7 mL of 1× PBS was added to neutralize the lysis reaction. Cells were filtered through a 50 μm CellTrics filter (Sysmex), spun again, and resuspended in fresh MACS buffer before counting the cells via 0.4% Trypan Blue solution (Gibco/ThermoFisher Scientific).

### 4.4 Isolation of Murine Bone Marrow HSPCs

Fresh bone marrow cells were enriched for HSPCs by negative cell selection of Lin‐ cells using magnetic cell sorting (MACS) with the mouse Direct Lineage Cell Depletion kit (Miltenyi Biotec, Cat #130‐110‐470). Cells and beads were added to a 50 μm CellTrics filter placed over an LS column (Miltenyi Biotec) in the magnetic field of a suitable MACS separator. Columns were washed 3× with 3 mL of MACS buffer. Unlabeled cells were counted using Trypan Blue, spun down, and kept on ice until antibody staining.

HSPCs were isolated by FACS sorting using the phenotypes as follows: (Lin‐ stands for CD3‐, B220‐, Gr1‐, NK1.1‐, Ter119‐)

**Table:** 

Name of cell population	Phenotype
LSK (Lin‐Sca1+kit+)	Lin‐Sca1+kit+
HSC (Hematopoietic Stem Cell)	Lin‐Sca1+kit+CD150+CD48‐
My‐HSC (Myeloid‐biased HSC)	Lin‐Sca1+kit+CD150hiCD48‐
Ly‐HSC (Lymphoid‐biased HSC)	Lin‐Sca1+kit+CD150loCD48‐

Cells were sorted into serum free mouse ATO culture medium and kept on ice. Cells for bulk ATOs were sorted into 1.5 mL Eppendorf tubes with 500 μL of ATO media, while cells for single cell ATOs were sorted into V‐bottom 96 well plates with 100 μL of ATO media per well. For single cell ATOs, 1 cell was sorted per well. Index sorting was used to record data about individual cells in Ly‐ and My‐HSC sorts. Positive control bulk ATO wells during single cell sorts had 25–50 cells per well.

### 4.5 Thymus Harvest

Mice were weighed and euthanized by high concentrations of isoflurane (Butler Schein, Dublin, OH) followed by cervical dislocation. Thymi were dissected and a single cell suspension was generated by gently mashing thymus tissue through a 50 μm cell strainer (Falcon). The cell stainer was rinsed with MACS buffer and cells were centrifuged at 300 g for 5 min at 4°C. Red blood cells were lysed with 1× AKC lysis buffer (Gibco/ThermoFisher Scientific) at room temperature for all studies (except cell cycling and apoptosis assays) as described above followed by centrifugation and resuspension in fresh MACS buffer. Cells were counted using Trypan Blue and kept on ice.

### 4.6 Isolation of Murine Thymus ETPs

Fresh thymocytes were enriched for double negative thymocytes by staining with anti‐CD4 PE and anti‐CD8 PE antibodies for 20 min at 4°C followed by negative selection of CD8+CD4+ cells by magnetic cell sorting (MACS) using Anti‐PE Microbeads UltraPure (Miltenyi Biotec, Cat #130‐105‐639). Cells and beads were added to a 50 μm Celltrics filter over an LS column in the magnetic field of a suitable MACS separator. Columns were washed 3× with 3 mL of MACS buffer. Unlabeled cells were counted using Trypan Blue, centrifuged, and kept on ice until FACS stain. Cells were first incubated at 4°C with anti‐kit (CD117) antibodies for 10–15 min before adding the rest of the antibody mix.

ETPs were isolated by FACS sorting using the phenotype Lin‐TCRβ‐CD3‐CD4‐CD8‐CD44+CD25‐kit^hi^ (Lin‐ stands for B220‐, CD11b‐, Gr1‐, Ter119‐).

### 4.7 Preparation of MS5‐mDLL4 for ATO Culture

Both freshly cultured and previously frozen MS5‐mDLL4 were used for ATO experiments. Fresh MS5‐mDLL4 cells were cultured in sterile filtered D10 (Dulbecco's Modification of Eagle's Medium (DMEM) (Cellgro, Cat #10‐017‐CV) 10% fetal bovine serum (FBS) (Gemini, Cat #900–208)) and harvested by trypsinization as previously described (Montel‐Hagen et al. 2020). Alternatively, frozen MS5‐mDLL4 aliquots were thawed and the cell pellet was resuspended in D10 on day 0 of ATO. MS5‐mDLL4 cells were centrifuged at 300 g for 5 min at 4°C. The cell pellet was resuspended in 10 mL of fresh D10. Cells were counted using Trypan Blue and kept on ice. We confirmed no difference in cell output or differentiation kinetics in ATOs with fresh versus frozen MS5‐mDLL4.

To freeze MS5‐mDLL4, MS5‐mDLL4 cell pellets (each with 10–20 M cells) were each combined with 1 mL of sterile filtered freezing media (90% FBS, 10% DMSO). Cells were flash frozen with liquid nitrogen before temporary storage at −80°C before transfer to liquid nitrogen for long‐term storage.

### 4.8 Preparation of ATO Culture Medium and Plates

Serum free mouse ATO medium is composed of DMEM‐F12 (GIBCO, Cat #11320033), 2% B27 supplement (ThermoFisher Scientific, Cat #17504–044), 30 μM L‐ascorbic acid 2‐phosphate sesquimagnesium salt hydrate (SigmaAldrich, Cat #A8960‐5G) reconstituted in 1× PBS, 1% penicillin/streptomycin (Gemini Bio‐Products, Cat #400‐109), and 1% Glutamax (ThermoFisher Scientific, Cat #35050‐061) (Montel‐Hagen et al. 2020). It can be stored for 1 month at 4°C. It is critical to use fresh preparations of ascorbic acid (i.e., 0.0087 g in 1 mL PBS for 1 L of media).

For complete mouse ATO medium, cytokines were freshly added to the serum free mouse ATO medium at each media change: 5 ng/mL rmFLT3L (Peprotech, Cat #250‐31 L), 5 ng/mL rmIL‐7 (Peprotech, Cat #217‐17 or R&D, Cat #407‐ML‐025/CF), 10 ng/mL rmSCF (Peprotech, Cat #250‐03) (SCF was added only for the first week of culture) and beta mercaptoethanol (bME) (0.05 mM) (Sigma‐Aldrich, Cat #M7522). We confirmed no difference in cell output, differentiation kinetics, or non‐T cell production with Peprotech versus R&D IL‐7.

0.4 μm Millicell transwell inserts (EMD Millipore, Cat #PICM0RG50) were placed in 6‐well plates, and 1 mL of complete mouse ATO medium was added per well. Plates were kept at 37°C.

### 4.9 Bulk ATO Cultures

Murine bulk ATOs were generated as previously described (Montel‐Hagen et al. 2020). MS5‐mDLL4 cells (150,000 cells/ATO) were combined with murine HSPC cells (25–100 cells per ATO) purified via FACS sorting and centrifuged at 300 g for 5 min at 4°C. Supernatants were carefully removed and the cell pellet was resuspended in an appropriate volume of mouse ATO media (5μL per ATO). 5 μL of the cell suspension was spread onto an insert in a 6‐well plate with media with 2 ATOs per insert. ATOs were kept in an incubator at 37°C with 5% CO_2_. Medium was changed completely every 3–4 days by aspiration from around the transwell insert followed by replacement with 1 mL of fresh complete mouse ATO medium.

### 4.10 Single Cell ATO Cultures

Murine single cell ATOs were generated with modifications from the original protocol (Montel‐Hagen et al. 2020) as described below. MS5‐mDLL4 cells were resuspended at a concentration of 150,000 cells per 50 μL in D10 medium and kept on ice.

After sorting, the 96‐well plates were centrifuged at 300 g for 5 min at 4°C to ensure each cell falls to the bottom of the well. 50 μL of the MS5‐mDLL4 cell suspension (150,000 cells per 50 μL) was added to each well of the 96 well plates containing one sorted cell per well using a multichannel and reservoir. Plates were kept on ice. Immediately before plating the ATOs, each 96‐well plate was centrifuged at 300 g for 5 min at 4°C. A manual multichannel was used to gently remove and discard approximately 150 μL of supernatant, leaving minimal liquid left with the cell pellet. To transfer the ATO cell pellet from a 96‐well plate to a 6‐well plate with transwell inserts, a multichannel with only two low‐attachment P20 pipet tips was used to add 10 μL of mouse ATO media to two wells at a time (10 μL/well), resuspend those cell pellets, and immediately spread all 10 μL of each ATO cell suspension onto an insert in a 6‐well plate with complete ATO media. This process was repeated for all cell pellets in the 96‐well plates, plating two ATOs at a time. Two ATOs were plated per insert, but harvested separately as described below. ATOs were kept in an incubator at 37°C with 5% CO_2_. Medium was changed completely every 3–4 days by aspiration from around the transwell insert followed by replacement with 1 mL of fresh complete mouse ATO medium.

### 4.11 Isolation of Thymocytes and T Cells

Bulk mouse ATO thymocytes were harvested by adding 1 mL MACS buffer to each transwell insert and briefly disaggregating the ATO by pipetting. All liquid was collected and passed through a 50 μm nylon CellTrics filter (Sysmex). Cells were centrifuged at 400 g for 5 min at 4°C, resuspended in fresh MACS buffer, and counted using Trypan Blue. No more than 2 M cells per sample were stained for flow cytometry.

For single cell mouse ATO harvests, 2 mL of MACS buffer was added to each well of a 12‐well plate (1 well/ATO to be harvested). Using a 1 mL pipet, one ATO was lifted from the insert and added to the well. The ATO was briefly disaggregated by pipetting, followed by passage through a 50 μm nylon CellTrics filter. This process was repeated for all ATOs. Approximately 32 ATOs were harvested per group per timepoint, and oftentimes harvests were distributed across 2–3 days due to the large number of ATOs harvested per day (up to 128 ATOs). Cells were then counted using either manual Trypan Blue count or automatic cell counter (Countess, ThermoFisher Scientific). If ATOs had greater than 100,000 cells/mL by Countess, samples would also be manually counted to compare for accuracy. No more than 2 M cells per sample were stained for flow cytometry in tubes to avoid cross‐contamination between high growth and low growth single cell ATOs. If there were > 500 K total cells, samples were also stained for a separate myeloid panel.

Flow cytometry analysis of thymic and ATO‐derived thymocyte populations used the following surface phenotypes: (Lin‐ stands here for: B220‐, NK1.1‐, CD11c‐, CD11b‐, Gr1‐, Ter119‐)

**Table:** 

Name of cell population	Surface Phenotype
ETP	CD45+Lin‐TCRβ‐CD3‐CD4‐CD8‐CD44+CD25‐kit^hi^
DN1	CD45+Lin‐TCRβ‐CD3‐CD4‐CD8‐CD44+CD25‐
DN2	CD45+Lin‐TCRβ‐CD3‐CD4‐CD8‐CD44+CD25+
DN3	CD45+Lin‐TCRβ‐CD3‐CD4‐CD8‐CD44‐CD25+
DN4	CD45+Lin‐TCRβ‐CD3‐CD4‐CD8‐CD44‐CD25‐
ISP8	CD45+Lin‐TCRβ‐CD3‐CD4‐CD8+
DP	CD45+Lin‐TCRβ‐CD3‐CD4+CD8+
DPl	CD45+Lin‐TCRβ+CD3+CD4+CD8+
SP4	CD45+Lin‐TCRβ+CD3+CD4+CD8‐
SP8	CD45+Lin‐TCRβ+CD3+CD4‐CD8+

Flow cytometry analysis of B, NK, red blood cell, and myeloid populations used the following surface phenotypes:

**Table:** 

Name of cell population	Phenotype
B	CD45+CD3‐CD4‐CD8‐B220+
NK	CD45+CD3‐CD4‐CD8‐NK1.1+
CD11c+	CD45+CD3‐CD4‐CD8‐CD11c+
CD11b+	CD45+CD3‐CD4‐CD8‐CD11b+
Gr1+	CD45+CD3‐CD4‐CD8‐Gr1+

### 4.12 Apoptosis and Cell Cycling Assays

Fresh thymocytes were isolated and enriched for DN as described above. Following extracellular staining, cells were fixed for 10 min in eBioscience 1× Fix/Perm buffer (ThermoFisher Scientific, Cat #88‐8824‐00) at room temperature in the dark; cells were then washed and stained intracellularly for Ki67, DAPI, and Caspase 3 for 30 min in eBioscience 1× Perm buffer at room temperature in the dark.

Flow cytometry analysis of thymocyte populations used the following surface and intracellular phenotypes (Lin‐ stands here for: B220‐, NK1.1‐, CD19‐, CD11b‐, Gr1, Ter119‐):

**Table:** 

Name of cell population	Surface phenotype
ETP	CD45+Lin‐CD3‐CD4‐CD8‐CD44+CD25‐kit^hi^
DN1	CD45+Lin‐CD3‐CD4‐CD8‐CD44+CD25—
DN2	CD45+Lin‐CD3‐CD4‐CD8‐CD44+CD25+
DN3	CD45+Lin‐CD3‐CD4‐CD8‐CD44‐CD25+
DN4	CD45+Lin‐CD3‐CD4‐CD8‐CD44‐CD25—

Name of cell subpopulation	Intracellular Phenotype
Live cells	Zombie R685‐ Caspase3‐
Early Apoptotic (Ap(E))	Zombie R685‐ Caspase3+
Late Apoptotic (Ap(L))	Zombie R685+ Caspase3+
Necrotic	Zombie NIR+ Caspase3‐
M	Ki67^hi^DAPI^hi^
G2	Ki67^mid^DAPI^hi^
S	Ki67+DAPI^mid^
G1	Ki67+DAPI^lo^
G0	Ki67‐DAPI^lo^
Ap(E) *(Ki67:DAPI assay)*	DAPI‐
Ap(L) *(Ki67:DAPI assay)*	Ki67‐DAPI^mid‐to‐hi^

### 4.13 Flow Cytometry

All extracellular flow cytometry stains were performed in MACS buffer for 30 min at 4°C or on ice in the dark. TruStain FcX (BioLegend, Cat #101320, Clone 93) was added to all samples for 5–10 min prior to antibody staining. DAPI (Life technologies, Cat #D1306) was added to samples prior to analysis. Analysis was performed on an LSRII Fortessa, Celesta, or spectral Cytek Aurora instruments. FACS sorting was performed on FACSARIA or FACSARIA‐H instruments (BD Biosciences) at the UCLA Broad Stem Cell Research Center Flow Cytometry Core. For all analyses except apoptosis and cell cycling assays, DAPI+ cells were gated out, and for all analyses single cells were gated based on FSC‐H versus FSC‐W/A and SSC‐H versus SSC‐W/A. For cell cycling experiments, doublets were included so as not to exclude mitotic cells. For apoptosis and cell cycling studies, DAPI was measured linearly; elsewhere it was measured logarithmically. Anti‐mouse antibody clones used for staining were obtained from BioLegend, BD, or ThermoFisher Scientific (Invitrogen) (Table S1).

In some single cell experiments, samples were analyzed using the Celesta automatic 96‐well plate reader. For high growth samples, approximately 100 μl of sample was diluted with 100 μL of MACS buffer per well. For low growth samples, 200 μL of sample was added per well. 150 μL of volume was analyzed at a flow rate between 1.5 μL/s and 3 μL/s. Two wells of water were run between high growth samples and 0–1 wells of water were run in between low growth samples. Flow cytometry data were analyzed with FlowJo software (Tree Star Inc).

### 4.14 Statistical Analysis

In all figures, n represents mice, individual cells, ATOs, or independent experiments and data are represented as mean ± standard deviation (SD) as indicated. Statistical analysis was performed using GraphPad Prism software and *p*‐values were calculated from the two‐tailed unpaired *t*‐test, multiple *t*‐test, one‐way ANOVA, or two‐way ANOVA. For all statistical analyses, only significant values are shown. A *p*‐value of < 0.05 was deemed significant (*p ≤ 0.05, **p ≤ 0.01, ***p ≤ 0.001, ****p ≤ 0.0001).

## Author Contributions

J.G. and G.M.C. conceptualized the project and designed the studies. J.G. performed the experiments and wrote the manuscript under supervision from G.M.C. J.L. performed laboratory work in support of the studies. E.R.M. performed cell cycling and apoptosis assays. V.S. performed preliminary studies important to the foundation of the project. S.C.B., E.R.M., G.Y., and X.Y. provided protocols and consultation for the project. K.D. contributed to experimental design and interpretation. G.M.C. oversaw the studies and provided funding. All authors reviewed, edited, and approved the manuscript.

## Funding

J.G. was supported by the Ruth L. Kirschstein National Research Service Award F30 (NIA F30AG086001) and the UCLA Tumor Cell Biology Training Program (NCI T32CA009056). J.G., E.R.M., and V.S. were supported by the UCLA‐Caltech Medical Scientist Training Program (NIGMS T32GM008042 and NIGMS T32GM152342). V.S. was also supported by a predoctoral fellowship from the BSCRC training program through generous support from the Rose Hills Foundation. G.Y. was supported by a Burroughs Wellcome Fund Career Award for Medical Scientists, and UCLA STAR Postdoctoral fellowship.

## Ethics Statement

All animal experiments were conducted in accordance with institutional and national guidelines and regulations for the care and use of laboratory animals. Ethical and legal approval was obtained prior to the start of the study under a protocol approved by the UCLA Chancellor's Animal Research Committee (ARC).

## Conflicts of Interest

G.M.C. is a co‐founder of Pluto Immunotherapeutics Inc., to which The Regents of University of California have licensed the intellectual property for development of the ATO model. G.M.C. is a co‐owner of the ATO intellectual property. The other authors declare no competing interests.

## Supporting information


**Figure S1:** Gating strategy of bone marrow for isolation of HSPCs and T cell differentiation in vitro. (a) Representative FACS plots of aged bone marrow for LSK and HSC sort. Gates are shown as a percentage of the parent population. Data are concatenated from six independent experiments into one FACS plot. (b) Representative FACS plots of young bone marrow for LSK and HSC sort. Gates are shown as a percentage of the parent population. Data are concatenated from six independent experiments into one FACS plot. (c) Representative FACS plots of early T cell differentiation in the ATO. Gates are shown as a percentage of the parent population.
**Figure S2:** Differentiation kinetics and population cell numbers of bulk LSK and HSC ATOs. (a) Cell numbers of subsets of DN cells at Week 1 of ATO initiated from bulk LSKs from young and aged male and female C57BL/6J mice, calculated using total cell numbers and frequency of live cells. Each dot represents two pooled ATOs. Error bar denotes ± SD (*n* = 48 ATOs total from two independent experiments, ordinary two‐way ANOVA). (b) Cell numbers of subsets at Week 3 of ATO initiated from bulk LSKs from young and aged male and female C57BL/6J mice calculated using total cell numbers and frequency of live cells. Each dot represents two pooled ATOs. Error bar denotes ± SD (*n* = 48 ATOs total from two independent experiments, ordinary two‐way ANOVA). (c) Frequencies of subsets at week 6 of ATO initiated from bulk LSKs from young and aged male and female C57BL/6J mice. Frequencies of DN cells, ISP8 cells, and DP cells are shown as a percentage of total live CD45+Lin‐ cells. Each dot represents two pooled ATOs. Error bar denotes ± SD (*n* = 48 ATOs total from two independent experiments, ordinary two‐way ANOVA). (d) Cell numbers of subsets at Week 6 of ATO initiated from bulk LSKs from young and aged male and female C57BL/6J mice, calculated using total cell numbers and frequency of live cells. Each dot represents two pooled ATOs. Error bar denotes ± SD (*n* = 48 ATOs total from two independent experiments, ordinary two‐way ANOVA). (e) Frequencies of subsets of DN cells at Week 1 of ATO initiated from bulk HSCs from young and aged male and female C57BL/6J mice, shown as a percentage of total DN cells. Each dot represents two pooled ATOs. Error bar denotes ± SD (*n* = 48 ATOs total from two independent experiments, ordinary two‐way ANOVA). (f) Cell numbers of subsets of DN cells at Week 1 of ATO initiated from bulk HSCs from young and aged male and female C57BL/6J mice calculated using total cell numbers and frequency of live cells. Each dot represents two pooled ATOs. Error bar denotes ± SD (*n* = 48 ATOs total from two independent experiments, ordinary two‐way ANOVA). (g) Cell numbers of subsets of DN cells at Week 3 of ATO initiated from bulk HSCs from young and aged male and female C57BL/6J mice, calculated using total cell numbers and frequency of live cells. Each dot represents two pooled ATOs. Error bar denotes ± SD (*n* = 48 ATOs total from two independent experiments, ordinary two‐way ANOVA). (h) Cell numbers of subsets at Week 6 of ATO initiated from bulk HSCs from young and aged male and female C57BL/6J mice, calculated using total cell numbers and frequency of live cells. Each dot represents two pooled ATOs. Error bar denotes ± SD (*n* = 48 ATOs total from two independent experiments, ordinary two‐way ANOVA). AF, aged female; AM, aged male; YF, young female; YM, young male. For all statistical analyses, only significant values are shown. A *p*‐value of < 0.05 was deemed significant (*p ≤ 0.05, **p ≤ 0.01, ***p ≤ 0.001, ****p ≤ 0.0001).
**Figure S3:** Myeloid cell numbers in bulk HSC ATOs and HSPC cell numbers and frequencies in young and aged bone marrow. (a) Cell numbers of B, NK, and myeloid cells at Week 3 of ATO initiated from bulk HSCs from young and aged male and female C57BL/6J mice, calculated using total cell numbers and frequency of live cells. Each dot represents two pooled ATOs. Error bar denotes ± SD (*n* = 48 ATOs total from two independent experiments, ordinary two‐way ANOVA). (b) Mean cell numbers of HSCs from bone marrow harvested from young (7–8 weeks old) and aged (18–24 months old) male and female C57BL/6J mice. Each dot represents an individual experiment with average of pooled data from 2 to 6 mice. Error bar denotes ± SD (*n* = 79 mice, ordinary one‐way ANOVA). (c) Mean cell numbers of LSKs from bone marrow harvested from young and aged male and female C57BL/6J mice. Each dot represents an individual experiment with average of pooled data from 2 to 6 mice. Error bar denotes ± SD (*n* = 79 mice, ordinary one‐way ANOVA). (d) Frequency of LSKs from young and aged male and female C57BL/6J Lin‐depleted mouse bone marrow, shown as a percentage of total live Lin‐depleted cells. Each dot represents an individual experiment with average of pooled data from 2 to 6 mice. Error bar denotes ± SD (*n* = 79 mice, ordinary one‐way ANOVA). (e) Frequency of HSCs from young and aged male and female C57BL/6J mouse bone marrow, shown as a percentage of LSKs. Each dot represents an individual experiment with average of pooled data from 2 to 6 mice. Error bar denotes ± SD (*n* = 79 mice, ordinary one‐way ANOVA). (f) Frequencies of Ly‐ and My‐HSCs from young and aged male and female C57BL/6J mouse bone marrow, shown as a percentage of LSKs. Each dot represents an individual experiment with average of pooled data from 2 to 6 mice. Error bar denotes ± SD (*n* = 79 mice, ordinary two‐way ANOVA). (g) Mean cell numbers of Ly‐ and My‐HSCs from bone marrow harvested from young and aged male and female C57BL/6J mice. Each dot represents an individual experiment with average of pooled data from 2 to 6 mice. Error bar denotes ± SD (*n* = 79 mice, ordinary two‐way ANOVA). AF, aged female; AM, aged male; YF, young female; YM, young male. For all statistical analyses, only significant values are shown. A *p*‐value of < 0.05 was deemed significant (*p ≤ 0.05, **p ≤ 0.01, ***p ≤ 0.001, ****p ≤ 0.0001).
**Figure S4:** Gating strategy of single Ly‐ and My‐HSC ATOs. (a) Representative FACS plots of single cell ATO with no growth. Gates are shown as a percentage of the parent population. (b) Representative FACS plots of single cell ATO with growth and T cell potential, as evidenced by expression of CD8, CD4, and the presence of DN3 cells. Gates are shown as a percentage of the parent population. (c) Representative FACS plots of single cell ATO without T cell potential. All cells are at DN1. Gates are shown as a percentage of the parent population.
**Figure S5:** Differentiation kinetics and population cell numbers of single Ly‐ and My‐HSC ATOs. (a) Frequencies of subsets at Week 4 of ATO initiated from single Ly‐ or My‐HSC from young and aged C57BL/6J mice, shown as a percentage of total live CD45+Lin‐ cells. Each dot represents one ATO. Error bar denotes ± SD (*n* = 234 ATOs total from six independent experiments, ordinary two‐way ANOVA). (b) Cell numbers of subsets at Week 4 of ATO initiated from single Ly‐ or My‐HSC from young and aged C57BL/6J mice, calculated using total cell numbers and frequency of live cells. Each dot represents one ATO. Error bar denotes ± SD (*n* = 234 ATOs total from six independent experiments, ordinary two‐way ANOVA). (c) Cell numbers of subsets of DN cells at Week 4 of ATO initiated from single Ly‐ or My‐HSC from young and aged C57BL/6J mice, calculated using total cell numbers and frequency of live cells. Each dot represents one ATO. Error bar denotes ± SD (*n* = 234 ATOs total from six independent experiments, ordinary two‐way ANOVA). (d) Cell numbers of subsets at Week 6 of ATO initiated from single Ly‐ or My‐HSC from young and aged C57BL/6J mice, calculated using total cell numbers and frequency of live cells. Each dot represents one ATO. Error bar denotes ± SD (*n* = 275 ATOs total from six independent experiments, ordinary two‐way ANOVA). (e) T cell potential at Week 6 of ATO initiated from single Ly‐ or My‐HSC from young and aged C57BL/6J mice, shown as a percentage of ATOs with growth. Age groups are shown separately. Each dot represents an individual experiment. Error bar denotes ± SD (*n* = 275 ATOs total from six independent experiments, ordinary one‐way ANOVA). (f) Frequencies of TCRβ+CD3+ cells at Week 6 of ATO initiated from single Ly‐ or My‐HSC from young and aged C57BL/6J mice, shown as a percentage of total live CD45+Lin‐ cells. Each dot represents one ATO. Error bar denotes ± SD (*n* = 275 ATOs total from six independent experiments, ordinary one‐way ANOVA). (g) Cell numbers of TCRβ+CD3+ cells at Week 6 of ATO initiated from single Ly‐ or My‐HSC from young and aged C57BL/6J mice, calculated using total cell numbers and frequency of live cells. Each dot represents one ATO. Error bar denotes ± SD (*n* = 275 ATOs total from six independent experiments, ordinary one‐way ANOVA). (h) Representative FACS plot of one ATO initiated from single Ly‐HSC with mature TCRβ+CD3+ subsets DP late (TCRβ+CD3+CD4+CD8+), SP4, and SP8. Frequencies are shown as a percentage of TCRβ+CD3+ cells. (i) Frequencies of DP late cells (TCRβ+CD3+CD4+CD8+) at Week 6 of ATO initiated from single Ly‐ or My‐HSC from young and aged C57BL/6J mice, shown as a percentage of TCRβ+CD3+ cells. Each dot represents one ATO. Error bar denotes ± SD (*n* = 275 ATOs total from six independent experiments, ordinary one‐way ANOVA). (j) Cell numbers of DP late cells (TCRβ+CD3+CD4+CD8+) at Week 6 of ATO initiated from single Ly‐ or My‐HSC from young and aged C57BL/6J mice, calculated using total cell numbers and frequency of live cells. Each dot represents one ATO. Error bar denotes ± SD (*n* = 275 ATOs total from six independent experiments, ordinary one‐way ANOVA). For all statistical analyses, only significant values are shown. A *p*‐value of < 0.05 was deemed significant (*p ≤ 0.05, **p ≤ 0.01, ***p ≤ 0.001, ****p ≤ 0.0001).
**Figure S6:** Myeloid cell production and lineage classification of ATOs initiated from single Ly‐ and My‐HSCs. (a) Frequencies of B, NK, and myeloid cells at Weeks 4 and 6 of ATO initiated from single Ly‐ or My‐HSC, shown as a percentage of total live CD45+CD3‐CD4‐CD8‐ cells. Age groups are shown separately. Each dot represents one ATO. Error bar denotes ± SD (*n* = 262 ATOs total from six independent experiments, ordinary two‐way ANOVA). (b) Representative FACS plots of single cell ATO classified as T only as evidenced by expression of CD8, CD4, and the presence of DN3 cells and less than 5% of Lin+cells. Gates are shown as a percentage of the parent population. (c) Representative FACS plots of single cell ATO classified as T/myeloid as evidenced by the presence of DN3 cells and > 5% of Lin+cells. Gates are shown as a percentage of the parent population. (d) Representative FACS plots of single cell ATO classified as myeloid only as evidenced by greater than 5% of Lin+cells and no evidence of T cell potential. Gates are shown as a percentage of the parent population. (e) Representative FACS plots of single cell ATO classified as indeterminate as evidenced by less than 5% of Lin+cells and no evidence of T cell potential. Gates are shown as a percentage of the parent population. (f) Frequencies of ATO lineage phenotypes at Week 4 of ATO initiated from single Ly‐ or My‐HSC, shown as a percentage of ATOs with growth. Age groups are shown separately. Each dot represents an individual experiment. Error bar denotes ± SD (*n* = 234 ATOs total from six independent experiments, ordinary two‐way ANOVA). (g) Frequencies of ATO lineage phenotypes at Week 6 of ATO initiated from single Ly‐ and My‐HSC, shown as a percentage of ATOs with growth. Age groups are shown separately. Each dot represents an individual experiment. Error bar denotes ± SD (*n* = 275 ATOs total from six independent experiments, ordinary two‐way ANOVA). For all statistical analyses, only significant values are shown. A *p*‐value of < 0.05 was deemed significant (*p ≤ 0.05, **p ≤ 0.01, ***p ≤ 0.001, ****p ≤ 0.0001).
**Figure S7:** ETP frequencies and gating strategy from young and aged thymus. (a) Frequency of ETPs from young and aged male and female C57BL/6J mouse thymus, shown as a percentage of DN1 cells. Each dot represents an individual experiment with average of pooled data from 2 to 6 mice. Error bar denotes ± SD (*n* = 54 mice, ordinary one‐way ANOVA). (b) Representative FACS plots of young thymus for ETP sort. Gates are shown as a percentage of the parent population. Data are concatenated from six independent experiments into one FACS plot. (c) Representative FACS plots of aged thymus for ETP sort. Gates are shown as a percentage of the parent population. Data sre concatenated from six independent experiments into one FACS plot. AF, aged female; AM, aged male; YF, young female; YM, young male. For all statistical analyses, only significant values are shown. A *p*‐value of < 0.05 was deemed significant (*p ≤ 0.05, **p ≤ 0.01, ***p ≤ 0.001, ****p ≤ 0.0001).
**Figure S8:** Differentiation kinetics and population cell numbers of single ETP ATOs. (a) Cell numbers at Week 6 of ATO initiated from single ETP from young and aged C57BL/6J mice. Each dot represents one ATO. Error bar denotes ± SD (*n* = 30 ATOs total from four independent experiments, Welch's *t*‐test). (b) Frequencies of subsets of DN cells at Week 3 of ATO initiated from single ETP from young and aged C57BL/6J mice, shown as a percentage of total DN cells. Each dot represents one ATO. Error bar denotes ± SD (*n* = 54 ATOs total from four independent experiments, multiple unpaired *t*‐test). (c) Frequencies of subsets at Week 6 of ATO initiated from single ETP from young and aged C57BL/6J mice. Frequencies of DN cells, ISP8 cells, and DP cells are shown as a percentage of total live CD45+Lin‐ cells. Each dot represents one ATO. Error bar denotes ± SD (*n* = 30 ATOs total from four independent experiments, multiple unpaired *t*‐test). (d) Cell numbers of subsets of DN cells at Week 2 of ATO initiated from single ETP from young and aged C57BL/6J mice, calculated using total cell numbers and frequency of live cells. Each dot represents one ATO. Error bar denotes ± SD (*n* = 46 ATOs total from four independent experiments, multiple unpaired *t*‐test). (e) Cell numbers of subsets at Week 2 of ATO initiated from single ETP from young and aged C57BL/6J mice, calculated using total cell numbers and frequency of live cells. Each dot represents one ATO. Error bar denotes ± SD (*n* = 46 ATOs total from four independent experiments, multiple unpaired *t*‐test). (f) Cell numbers of subsets of DN cells at Week 3 of ATO initiated from single ETP from young and aged C57BL/6J mice, calculated using total cell numbers and frequency of live cells. Each dot represents one ATO. Error bar denotes ± SD (*n* = 54 total ATOs from four independent experiments, multiple unpaired *t*‐test). (g) Cell numbers of subsets at Week 3 of ATO initiated from single ETP from young and aged C57BL/6J mice, calculated using total cell numbers and frequency of live cells. Each dot represents one ATO. Error bar denotes ± SD (*n* = 54 total ATOs from four independent experiments, multiple unpaired *t*‐test). (h) Cell numbers of subsets of DN cells at Week 6 of ATO initiated from single ETP from young and aged C57BL/6J mice, calculated using total cell numbers and frequency of live cells. Each dot represents one ATO. Error bar denotes ± SD (*n* = 30 total ATOs from four independent experiments, multiple unpaired *t*‐test). (i) Cell numbers of subsets at Week 6 of ATO initiated from single ETP from young and aged C57BL/6J mice, calculated using total cell numbers and frequency of live cells. Each dot represents one ATO. Error bar denotes ± SD (*n* = 30 ATOs total from four independent experiments, multiple unpaired *t*‐test). For all statistical analyses, only significant values are shown. A *p*‐value of < 0.05 was deemed significant (*p ≤ 0.05, **p ≤ 0.01, ***p ≤ 0.001, ****p ≤ 0.0001)
**Figure S9:** Differentiation kinetics and population cell numbers of mature cell subsets generated from single ETPs at week 3 of ATO. (a) Frequencies of TCRβ+CD3+ cells initiated from single ETP from young and aged C57BL/6J mice, shown as a percentage of total live CD45+Lin‐ cells. Each dot represents one ATO. Error bar denotes ± SD (*n* = 54 ATOs total from four independent experiments, Welch's *t*‐test). (b) Cell numbers of TCRβ+CD3+ cells initiated from single ETP from young and aged C57BL/6J mice, calculated using total cell numbers and frequency of live cells. Each dot represents one ATO. Error bar denotes ± SD (*n* = 54 total ATOs from four independent experiments, Welch's *t*‐test). (c) Representative FACS plot of one ATO initiated from single aged ETP with mature TCRβ+CD3+ subsets DP late (TCRβ+CD3+CD4+CD8+), SP4, and SP8. Frequencies are shown as a percentage of TCRβ+CD3+ cells. (d) Frequencies of DP late cells (TCRβ+CD3+CD4+CD8+) initiated from single ETP from young and aged C57BL/6J mice, shown as a percentage of TCRβ+CD3+ cells. Each dot represents one ATO. Error bar denotes ± SD (*n* = 54 ATOs total from four independent experiments, Welch's *t*‐test). (e) Cell numbers of DP late cells (TCRβ+CD3+CD4+CD8+) initiated from single ETP from young and aged C57BL/6J mice, calculated using total cell numbers and frequency of live cells. Each dot represents one ATO. Error bar denotes ± SD (*n* = 54 total ATOs from four independent experiments, Welch's *t*‐test). For all statistical analyses, only significant values are shown. A *p*‐value of < 0.05 was deemed significant (*p ≤ 0.05, **p ≤ 0.01, ***p ≤ 0.001, ****p ≤ 0.0001).
**Figure S10:** Apoptosis and cell cycling of early thymocytes from aged and young thymus. (a) Representative FACS plots of cell populations in Zombie:Caspase 3 apoptosis assay from young thymus. Gates are shown as a percentage of the parent population. (b) Representative FACS plots of cell populations in DAPI:Ki67 cell cycling and apoptosis assay from young thymus. Gates are shown as a percentage of the parent population. (c) Frequencies of early (Ap(E)) and late (Ap(L)) apoptotic cells from young and aged thymus using Zombie:Caspase 3 assay, shown as a percentage of the corresponding thymocyte subset. Error bar denotes ± SD (*n* = 13 total mice from three independent experiments, multiple unpaired *t*‐test). (d) Frequencies of early (Ap(E)) and late (Ap(L)) apoptotic cells from young and aged thymus using DAPI:Ki67 assay, shown as a percentage of the corresponding thymocyte subset. Error bar denotes ± SD (*n* = 16 total mice from three independent experiments, multiple unpaired *t*‐test). (e) Frequencies of proliferating cells in G2, S, and M phase from young and aged thymus, shown as a percentage of the corresponding thymocyte subset. Error bar denotes ± SD (*n* = 16 total mice from three independent experiments, multiple unpaired *t*‐test). For all statistical analyses, only significant values are shown. A *p*‐value of < 0.05 was deemed significant (*p ≤ 0.05, **p ≤ 0.01, ***p ≤ 0.001, ****p ≤ 0.0001).
**Table S1:** Anti‐mouse antibodies. Antibodies are listed based on relevant sort or developmental panel and include the name, fluorophore, source, catalog number, and clone.

## Data Availability

Data will be made available upon request.
