# Supplementary material for: Clonal Analyses Reveal the Impact of Hematopoietic Stem and Progenitor Cell Aging on T Cell Development
Source: Aging Cell. 2026 Jun 29;25(7):e70615. doi: 10.1111/acel.70615 (PMC13314711; doi:10.1111/acel.70615)
Supplement: Supplementary file 1 — Figure S1: Gating strategy of bone marrow for isolation of HSPCs and T cell differentiation in vitro. (a) Representative FACS plots of aged bone marrow for LSK and HSC sort. Gates are shown as a percentage of the parent population. Data are concatenated from six independent experiments into one FACS plot. (b) Representative FACS plots of young bone marrow for LSK and HSC sort. Gates are shown as a percentage of the parent population. Data are concatenated from six independent experiments into one FACS plot. (c) Representative FACS plots of early T cell differentiation in the ATO. Gates are shown as a percentage of the parent population. Figure S2: Differentiation kinetics and population cell numbers of bulk LSK and HSC ATOs. (a) Cell numbers of subsets of DN cells at Week 1 of ATO initiated from bulk LSKs from young and aged male and female C57BL/6J mice, calculated using total cell numbers and frequency of live cells. Each dot represents two pooled ATOs. Error bar denotes ± SD (n = 48 ATOs total from two independent experiments, ordinary two‐way ANOVA). (b) Cell numbers of subsets at Week 3 of ATO initiated from bulk LSKs from young and aged male and female C57BL/6J mice calculated using total cell numbers and frequency of live cells. Each dot represents two pooled ATOs. Error bar denotes ± SD (n = 48 ATOs total from two independent experiments, ordinary two‐way ANOVA). (c) Frequencies of subsets at week 6 of ATO initiated from bulk LSKs from young and aged male and female C57BL/6J mice. Frequencies of DN cells, ISP8 cells, and DP cells are shown as a percentage of total live CD45+Lin‐ cells. Each dot represents two pooled ATOs. Error bar denotes ± SD (n = 48 ATOs total from two independent experiments, ordinary two‐way ANOVA). (d) Cell numbers of subsets at Week 6 of ATO initiated from bulk LSKs from young and aged male and female C57BL/6J mice, calculated using total cell numbers and frequency of live cells. Each dot represents two pooled ATOs. Error bar denotes [file ACEL-25-e70615-s001.pdf]

Supplementary Materials for

Clonal analyses reveal the impact of hematopoietic stem and progenitor cell aging on T cell development

Julia Gensheimer[1,2], Jessica LaGosh[2], Emma R. Moulton[1,2], Victoria Sun[2], Stephanie C. de Barros[2], Encarnacion Montecino-Rodriguez[2], Gloria Yiu[3], Xuegang Yuan[2], Kenneth Dorshkind[2], Gay M. Crooks[2,4,5,6,7,8]

1. Molecular Biology Interdepartmental Program, University of California, Los Angeles, Los Angeles, CA 90095, USA
2. Department of Pathology and Laboratory Medicine, David Geffen School of Medicine, University of California, Los Angeles, Los Angeles, CA 90095, USA
3. Division of Rheumatology, Department of Medicine, David Geffen School of Medicine, University of California, Los Angeles, Los Angeles, CA 90095, USA
4. Molecular Biology Institute, University of California, Los Angeles, Los Angeles, CA 90095, USA
5. Broad Stem Cell Research Center, David Geffen School of Medicine, University of California, Los Angeles, Los Angeles, CA 90095, USA
6. Jonsson Comprehensive Cancer Center, David Geffen School of Medicine, University of California, Los Angeles, Los Angeles, CA 90095, USA
7. Department of Pediatrics, David Geffen School of Medicine, University of California, Los Angeles, Los Angeles, CA 90095, USA
8. Corresponding Author, gcrooks@mednet.ucla.edu

This file includes:

Supplemental Figure 1: Gating strategy of bone marrow for isolation of HSPCs and T cell differentiation *in vitro*.
Supplemental Figure 2: Differentiation kinetics and population cell numbers of bulk LSK and HSC ATOs.
Supplemental Figure 3: Myeloid cell numbers in bulk HSC ATOs and HSPC cell numbers and frequencies in young and aged bone marrow.
Supplemental Figure 4: Gating strategy of single Ly- and My-HSC ATOs.
Supplemental Figure 5: Differentiation kinetics and population cell numbers of single Ly- and My-HSC ATOs.
Supplemental Figure 6: Myeloid cell production and lineage classification of ATOs initiated from single Ly- and My-HSCs.
Supplemental Figure 7: ETP frequencies and gating strategy from young and aged thymus.
Supplemental Figure 8. Differentiation kinetics and population cell numbers of single ETP ATOs.
Supplemental Figure 9. Differentiation kinetics and population cell numbers of mature cell subsets generated from single ETPs at week 3 of ATO.
Supplemental Figure 10. Apoptosis and cell cycling of early thymocytes from aged and young thymus.
Supplemental Table 1: Anti-mouse antibodies.

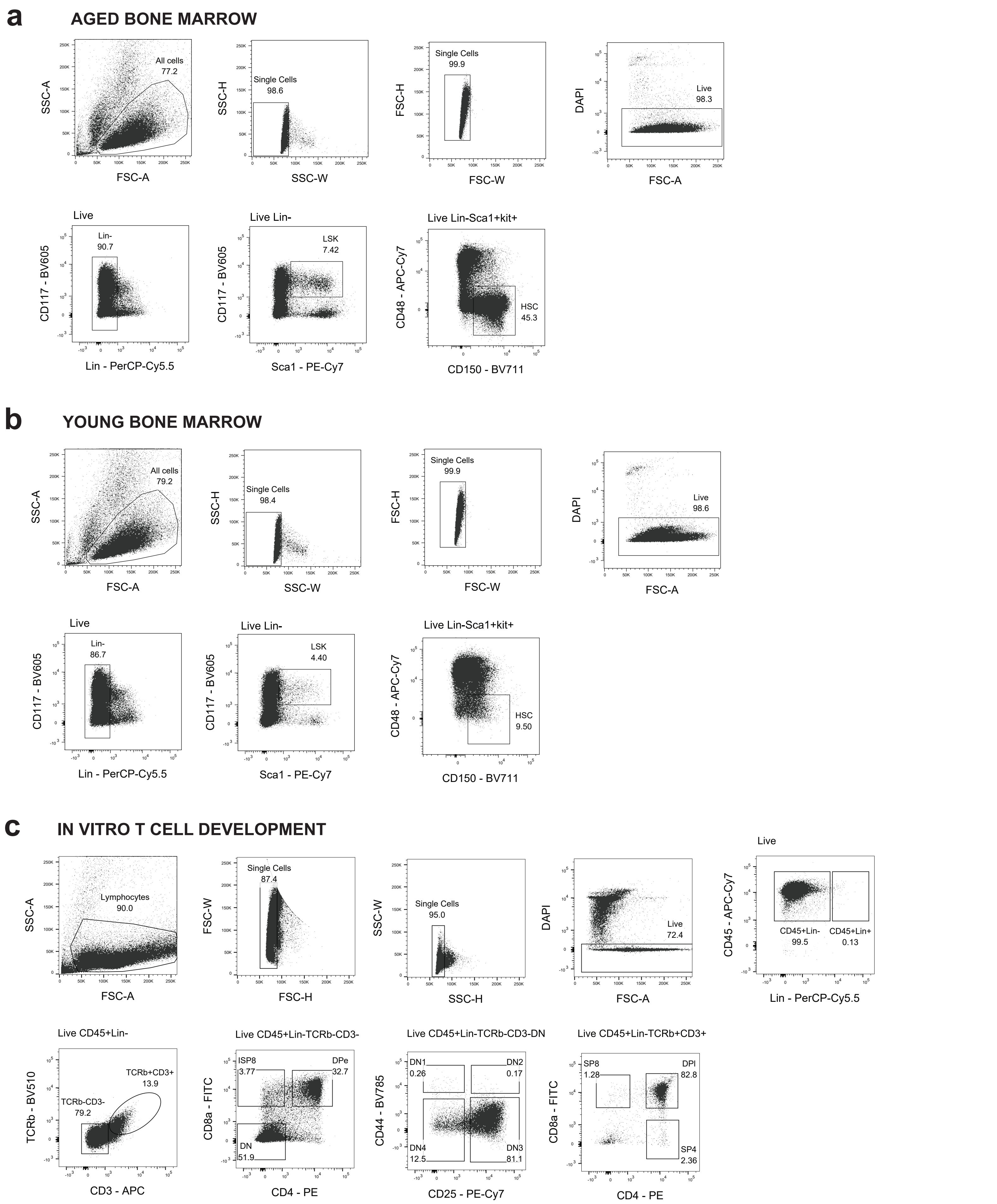

Supplemental Figure 1

Supplemental Figure 1. Gating strategy of bone marrow for isolation of HSPCs and T cell differentiation *in vitro*.

a) Representative FACS plots of aged bone marrow for LSK and HSC sort. Gates are shown as a percentage of the parent population. Data is concatenated from six independent experiments into one FACS plot. **b)** Representative FACS plots of young bone marrow for LSK and HSC sort. Gates are shown as a percentage of the parent population. Data is concatenated from six independent experiments into one FACS plot. **c)** Representative FACS plots of early T cell differentiation in the ATO. Gates are shown as a percentage of the parent population.

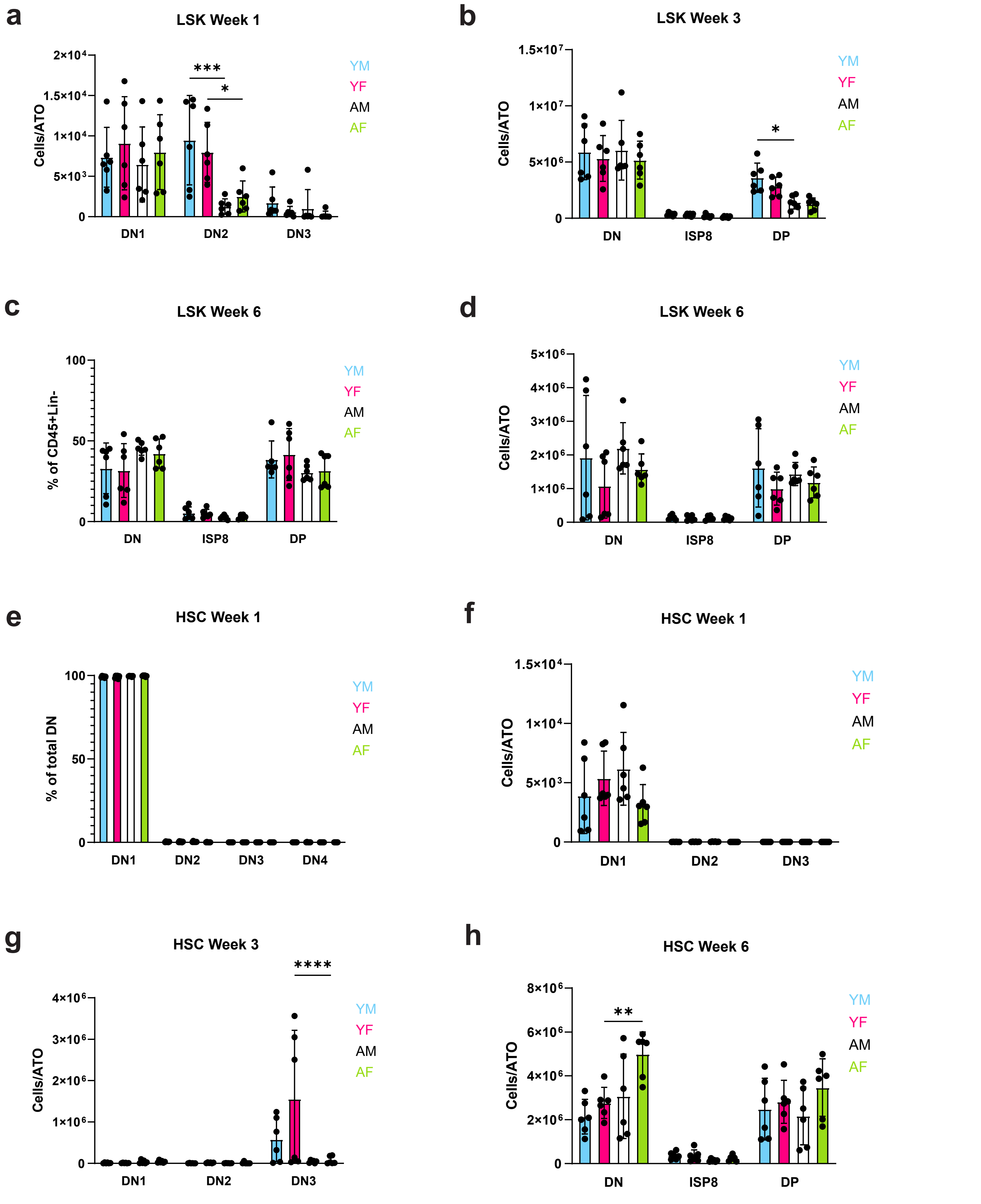

Supplemental Figure 2

Supplemental Figure 2. Differentiation kinetics and population cell numbers of bulk LSK and HSC ATOs.

a) Cell numbers of subsets of DN cells at week 1 of ATO initiated from bulk LSKs from young and aged male and female C57BL/6J mice, calculated using total cell numbers and frequency of live cells. Each dot represents 2 pooled ATOs. Error bar denotes +/- SD (n = 48 ATOs total from 2 independent experiments, ordinary two-way ANOVA). **b)** Cell numbers of subsets at week 3 of ATO initiated from bulk LSKs from young and aged male and female C57BL/6J mice, calculated using total cell numbers and frequency of live cells. Each dot represents 2 pooled ATOs. Error bar denotes +/- SD (n = 48 ATOs total from 2 independent experiments, ordinary two-way ANOVA). **c)** Frequencies of subsets at week 6 of ATO initiated from bulk LSKs from young and aged male and female C57BL/6J mice. Frequencies of DN cells, ISP8 cells, and DP cells are shown as a percentage of total live CD45+Lin- cells. Each dot represents 2 pooled ATOs. Error bar denotes +/- SD (n = 48 ATOs total from 2 independent experiments, ordinary two-way ANOVA). **d)** Cell numbers of subsets at week 6 of ATO initiated from bulk LSKs from young and aged male and female C57BL/6J mice, calculated using total cell numbers and frequency of live cells. Each dot represents 2 pooled ATOs. Error bar denotes +/- SD (n = 48 ATOs total from 2 independent experiments, ordinary two-way ANOVA). **e)** Frequencies of subsets of DN cells at week 1 of ATO initiated from bulk HSCs from young and aged male and female C57BL/6J mice, shown as a percentage of total DN cells. Each dot represents 2 pooled ATOs. Error bar denotes +/- SD (n = 48 ATOs total from 2 independent experiments, ordinary two-way ANOVA). **f)** Cell numbers of subsets of DN cells at week 1 of ATO initiated from bulk HSCs from young and aged male and female C57BL/6J mice, calculated using total cell numbers and frequency of live cells. Each dot represents 2 pooled ATOs. Error bar denotes +/- SD (n = 48 ATOs total from 2 independent experiments, ordinary two-way ANOVA). **g)** Cell numbers of subsets of DN cells at week 3 of ATO initiated from bulk HSCs from young and aged male and female C57BL/6J mice, calculated using total cell numbers and frequency of live cells. Each dot represents 2 pooled ATOs. Error bar denotes +/- SD (n = 48 ATOs total from 2 independent experiments, ordinary two-way ANOVA). **h)** Cell numbers of subsets at week 6 of ATO initiated from bulk HSCs from young and aged male and female C57BL/6J mice, calculated using total cell numbers and frequency of live cells. Each dot represents 2 pooled ATOs. Error bar denotes +/- SD (n = 48 ATOs total from 2 independent experiments, ordinary two-way ANOVA).

YM = young male; YF = young female; AM = aged male; AF = aged female. For all statistical analyses, only significant values are shown. A p value of <0.05 was deemed significant ($p^* \leq 0.05$, $^{**}p \leq 0.01$, $^{***}p \leq 0.001$, $^{****}p \leq 0.0001$).

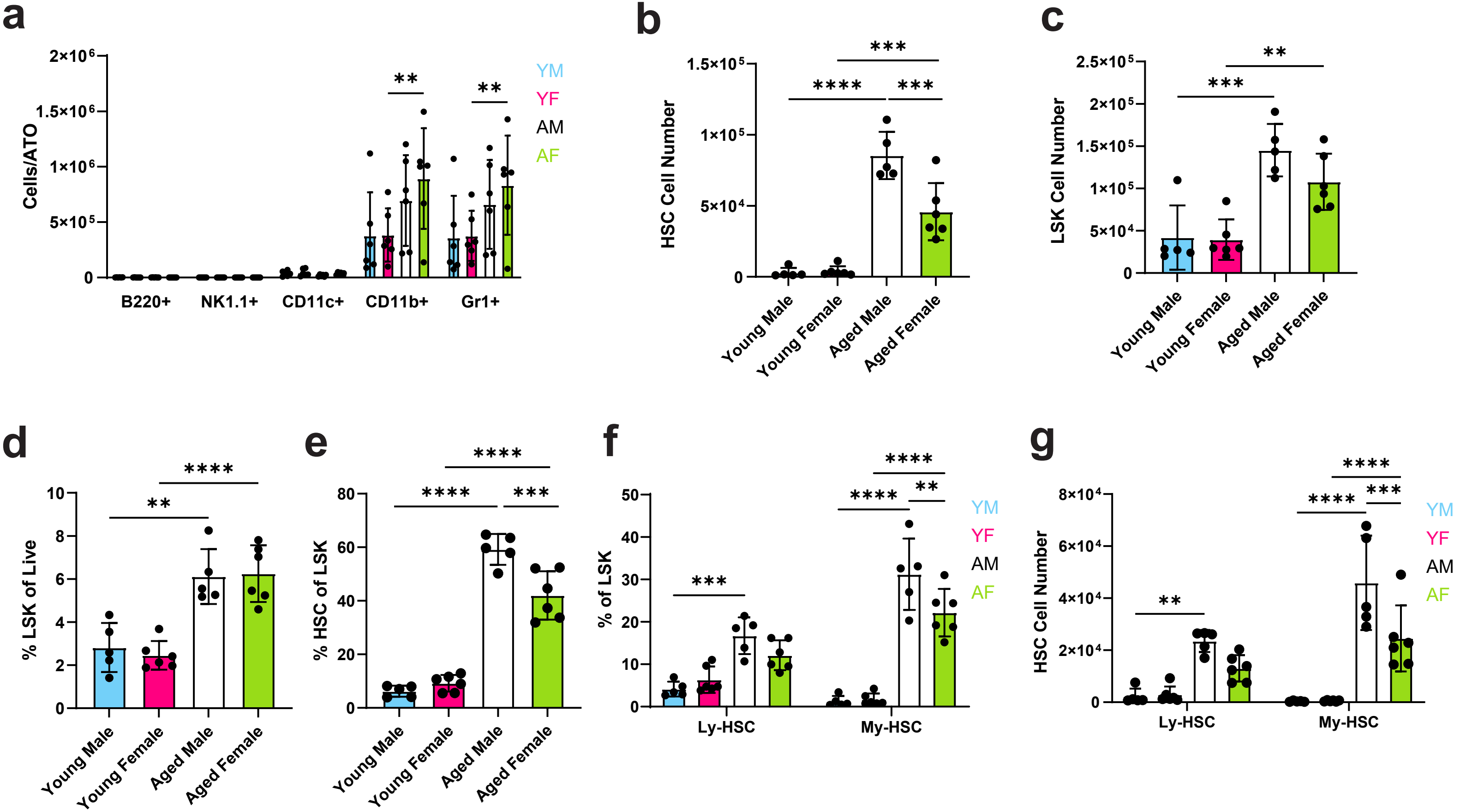

Supplemental Figure 3

Supplemental Figure 3. Myeloid cell numbers in bulk HSC ATOs and HSPC cell numbers and frequencies in young and aged bone marrow.

a) Cell numbers of B, NK, and myeloid cells at week 3 of ATO initiated from bulk HSCs from young and aged male and female C57BL/6J mice, calculated using total cell numbers and frequency of live cells. Each dot represents 2 pooled ATOs. Error bar denotes +/- SD (n = 48 ATOs total from 2 independent experiments, ordinary two-way ANOVA). **b)** Mean cell numbers of HSCs from bone marrow harvested from young (7-8 weeks old) and aged (18-24 months old) male and female C57BL/6J mice. Each dot represents an individual experiment with average of pooled data from 2-6 mice. Error bar denotes +/- SD (n = 79 mice, ordinary one-way ANOVA). **c)** Mean cell numbers of LSKs from bone marrow harvested from young and aged male and female C57BL/6J mice. Each dot represents an individual experiment with average of pooled data from 2-6 mice. Error bar denotes +/- SD (n = 79 mice, ordinary one-way ANOVA). **d)** Frequency of LSKs from young and aged male and female C57BL/6J Lin-depleted mouse bone marrow, shown as a percentage of total live Lin-depleted cells. Each dot represents an individual experiment with average of pooled data from 2-6 mice. Error bar denotes +/- SD (n = 79 mice, ordinary one-way ANOVA). **e)** Frequency of HSCs from young and aged male and female C57BL/6J mouse bone marrow, shown as a percentage of LSKs. Each dot represents an individual experiment with average of pooled data from 2-6 mice. Error bar denotes +/- SD (n = 79 mice, ordinary one-way ANOVA). **f)** Frequencies of Ly- and My-HSCs from young and aged male and female C57BL/6J mouse bone marrow, shown as a percentage of LSKs. Each dot represents an individual experiment with average of pooled data from 2-6 mice. Error bar denotes +/- SD (n = 79 mice, ordinary two-way ANOVA). **g)** Mean cell numbers of Ly- and My-HSCs from bone marrow harvested from young and aged male and female C57BL/6J mice. Each dot represents an individual experiment with average of pooled data from 2-6 mice. Error bar denotes +/- SD (n = 79 mice, ordinary two-way ANOVA).

YM = young male; YF = young female; AM = aged male; AF = aged female. For all statistical analyses, only significant values are shown. A p value of <0.05 was deemed significant (p* ≤ 0.05, **p ≤ 0.01, ***p ≤ 0.001, ****p ≤ 0.0001).

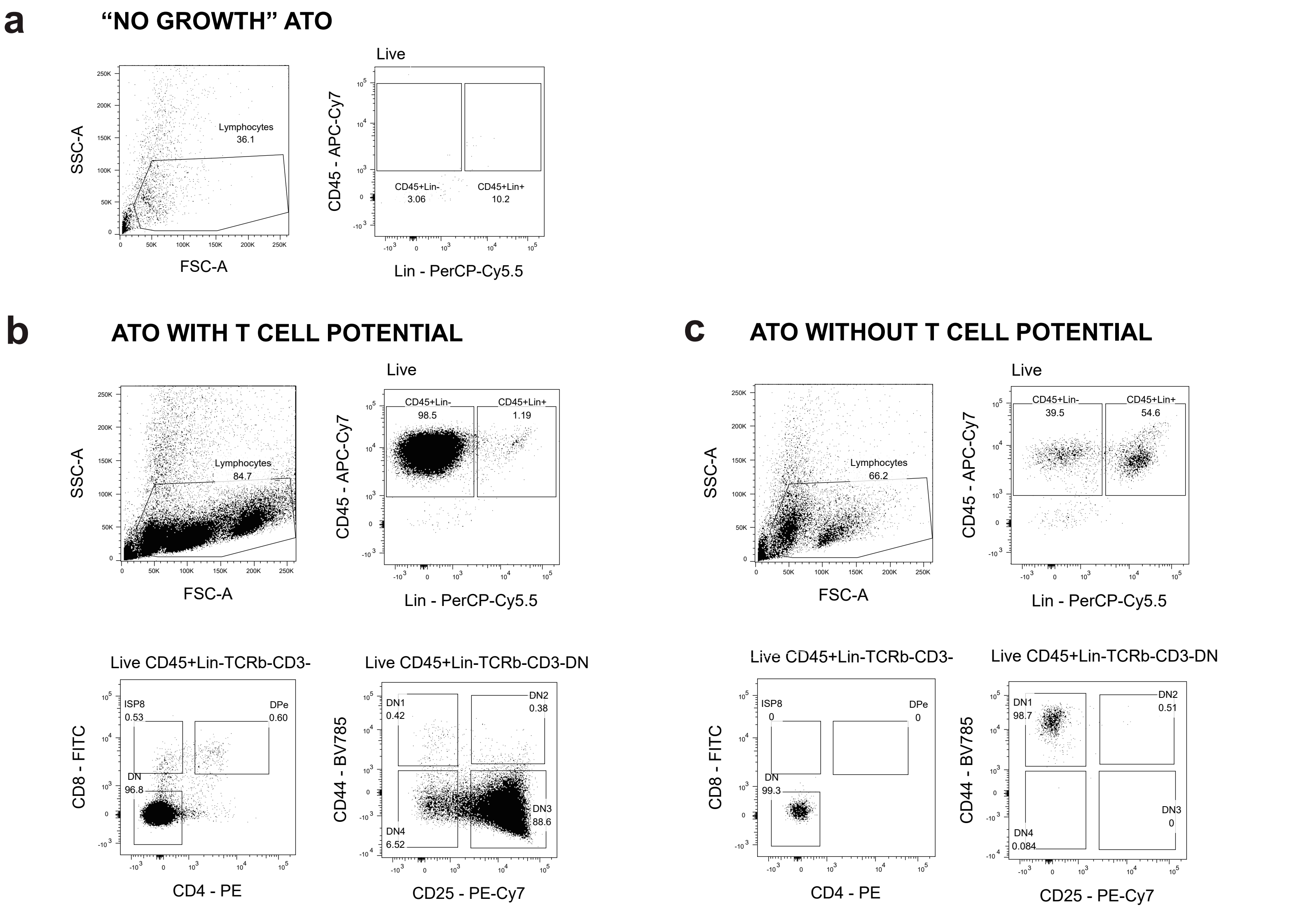

Supplemental Figure 4

Supplemental Figure 4. Gating strategy of single Ly- and My-HSC ATOs.

a) Representative FACS plots of single cell ATO with no growth. Gates are shown as a percentage of the parent population. **b)** Representative FACS plots of single cell ATO with growth and T cell potential, as evidenced by expression of CD8, CD4, and the presence of DN3 cells. Gates are shown as a percentage of the parent population. **c)** Representative FACS plots of single cell ATO without T cell potential. All cells are at DN1. Gates are shown as a percentage of the parent population.

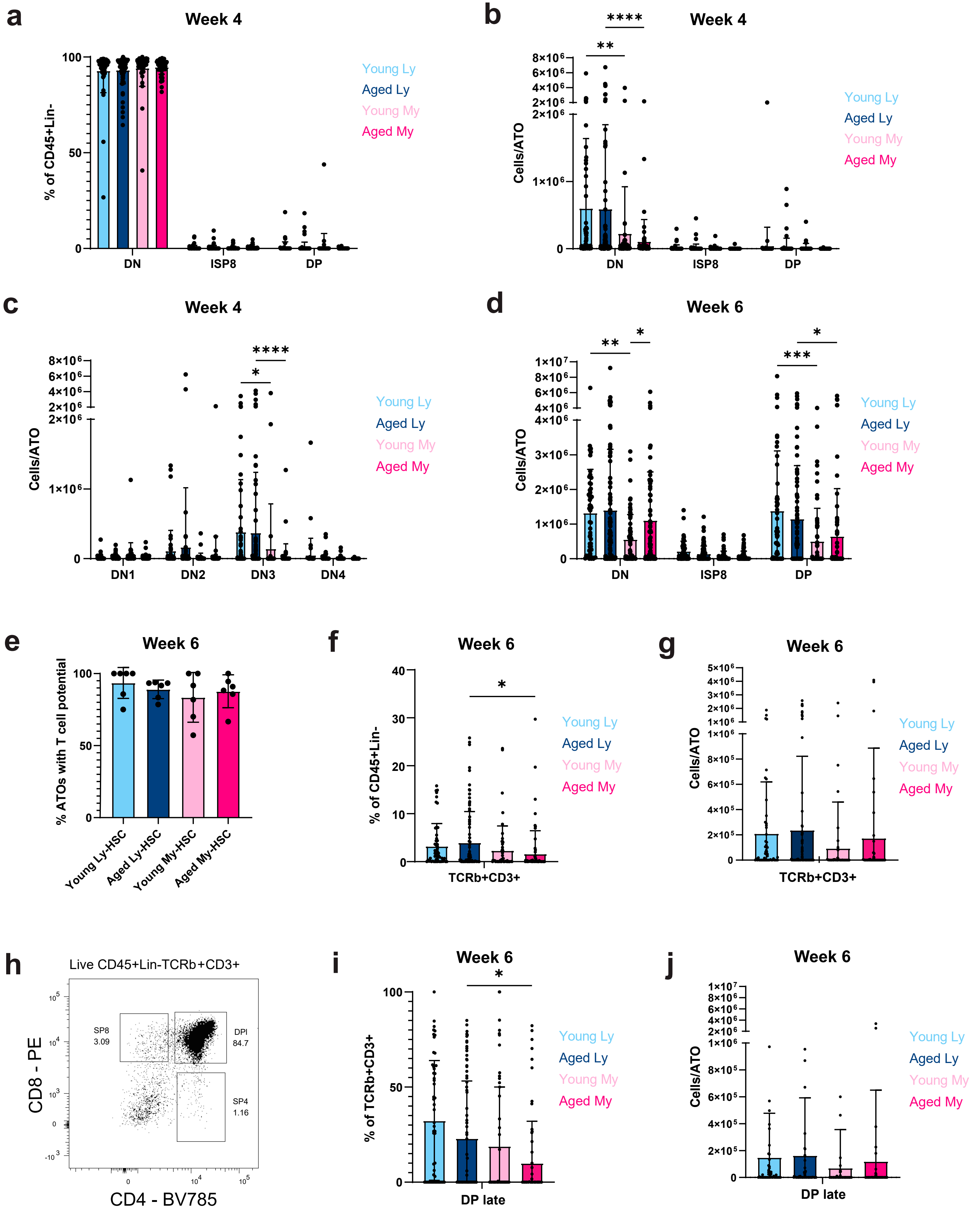

Supplemental Figure 5

Supplemental Figure 5. Differentiation kinetics and population cell numbers of single Ly- and My-HSC ATOs.

a) Frequencies of subsets at week 4 of ATO initiated from single Ly- or My-HSC from young and aged C57BL/6J mice, shown as a percentage of total live CD45+Lin- cells. Each dot represents 1 ATO. Error bar denotes +/- SD (n = 234 ATOs total from 6 independent experiments, ordinary two-way ANOVA). **b)** Cell numbers of subsets at week 4 of ATO initiated from single Ly- or My-HSC from young and aged C57BL/6J mice, calculated using total cell numbers and frequency of live cells. Each dot represents 1 ATO. Error bar denotes +/- SD (n = 234 ATOs total from 6 independent experiments, ordinary two-way ANOVA). **c)** Cell numbers of subsets of DN cells at week 4 of ATO initiated from single Ly- or My-HSC from young and aged C57BL/6J mice, calculated using total cell numbers and frequency of live cells. Each dot represents 1 ATO. Error bar denotes +/- SD (n = 234 ATOs total from 6 independent experiments, ordinary two-way ANOVA). **d)** Cell numbers of subsets at week 6 of ATO initiated from single Ly- or My-HSC from young and aged C57BL/6J mice, calculated using total cell numbers and frequency of live cells. Each dot represents 1 ATO. Error bar denotes +/- SD (n = 275 ATOs total from 6 independent experiments, ordinary two-way ANOVA). **e)** T cell potential at week 6 of ATO initiated from single Ly- or My-HSC from young and aged C57BL/6J mice, shown as a percentage of ATOs with growth. Age groups are shown separately. Each dot represents an individual experiment. Error bar denotes +/- SD (n = 275 ATOs total from 6 independent experiments, ordinary one-way ANOVA). **f)** Frequencies of TCRβ+CD3+ cells at week 6 of ATO initiated from single Ly- or My-HSC from young and aged C57BL/6J mice, shown as a percentage of total live CD45+Lin- cells. Each dot represents 1 ATO. Error bar denotes +/- SD (n = 275 ATOs total from 6 independent experiments, ordinary one-way ANOVA). **g)** Cell numbers of TCRβ+CD3+ cells at week 6 of ATO initiated from single Ly- or My-HSC from young and aged C57BL/6J mice, calculated using total cell numbers and frequency of live cells. Each dot represents 1 ATO. Error bar denotes +/- SD (n = 275 ATOs total from 6 independent experiments, ordinary one-way ANOVA). **h)** Representative FACS plot of one ATO initiated from single Ly-HSC with mature TCRβ+CD3+ subsets DP late (TCRβ+CD3+CD4+CD8+), SP4, and SP8. Frequencies are shown as a percentage of TCRβ+CD3+ cells. **i)** Frequencies of DP late cells (TCRβ+CD3+CD4+CD8+) at week 6 of ATO initiated from single Ly- or My-HSC from young and aged C57BL/6J mice, shown as a percentage of TCRβ+CD3+ cells. Each dot represents 1 ATO. Error bar denotes +/- SD (n = 275 ATOs total from 6 independent experiments, ordinary one-way ANOVA). **j)** Cell numbers of DP late cells (TCRβ+CD3+CD4+CD8+) at week 6 of ATO initiated from single Ly- or My-HSC from young and aged C57BL/6J mice, calculated using total cell numbers and frequency of live cells. Each dot represents 1 ATO. Error bar denotes +/- SD (n = 275 ATOs total from 6 independent experiments, ordinary one-way ANOVA).

For all statistical analyses, only significant values are shown. A p value of <0.05 was deemed significant ($p^* \leq 0.05$, $^{**}p \leq 0.01$, $^{***}p \leq 0.001$, $^{****}p \leq 0.0001$).

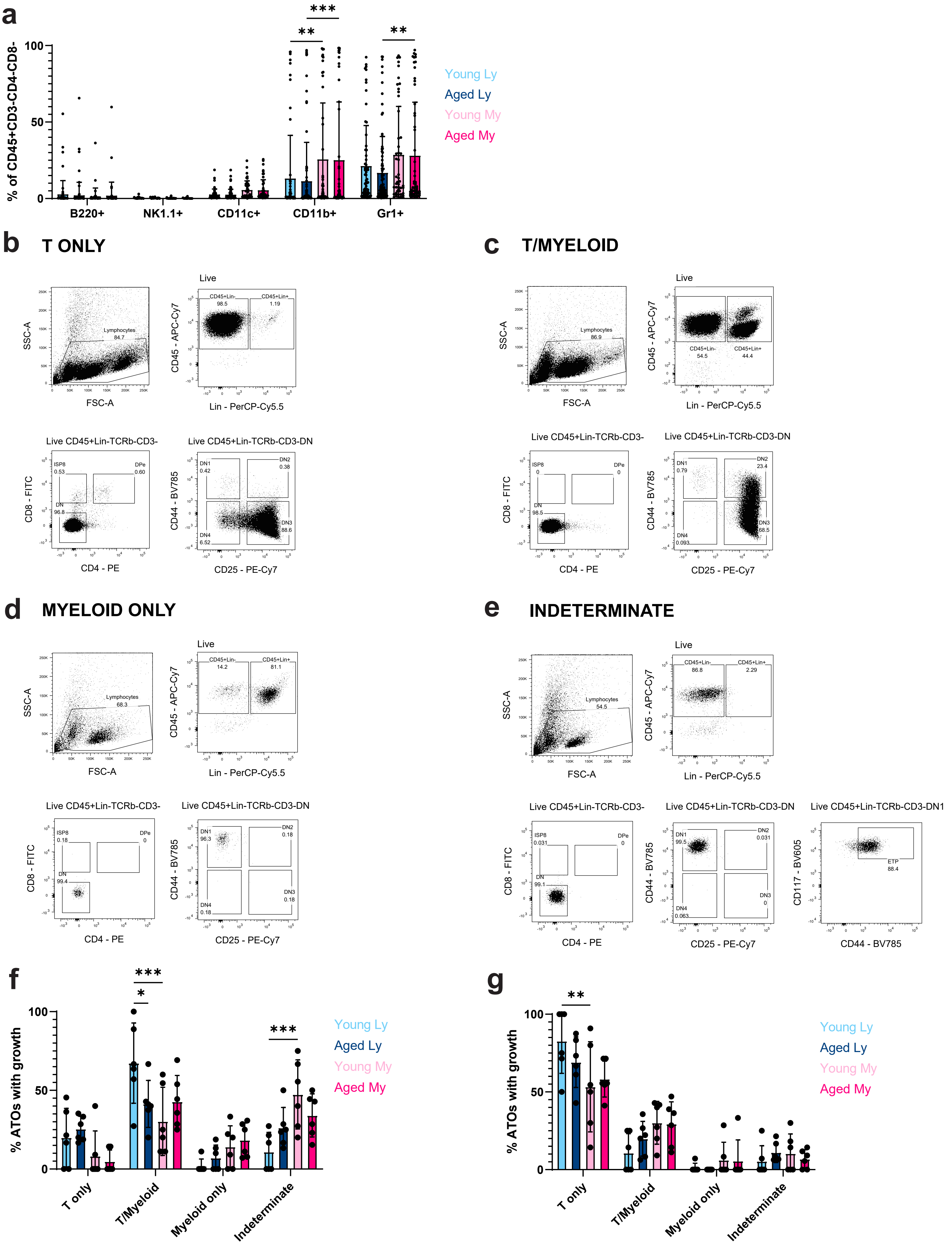

Supplemental Figure 6

Supplemental Figure 6. Myeloid cell production and lineage classification of ATOs initiated from single Ly- and My-HSCs.

a) Frequencies of B, NK, and myeloid cells at weeks 4 and 6 of ATO initiated from single Ly- or My-HSC, shown as a percentage of total live CD45+CD3-CD4-CD8- cells. Age groups are shown separately. Each dot represents 1 ATO. Error bar denotes +/- SD (n = 262 ATOs total from 6 independent experiments, ordinary two-way ANOVA). **b)** Representative FACS plots of single cell ATO classified as T only as evidenced by expression of CD8, CD4, and the presence of DN3 cells and less than 5% of Lin+ cells. Gates are shown as a percentage of the parent population. **c)** Representative FACS plots of single cell ATO classified as T/myeloid as evidenced by the presence of DN3 cells and greater than 5% of Lin+ cells. Gates are shown as a percentage of the parent population. **d)** Representative FACS plots of single cell ATO classified as myeloid only as evidenced by greater than 5% of Lin+ cells and no evidence of T cell potential. Gates are shown as a percentage of the parent population. **e)** Representative FACS plots of single cell ATO classified as indeterminate as evidenced by less than 5% of Lin+ cells and no evidence of T cell potential. Gates are shown as a percentage of the parent population. **f)** Frequencies of ATO lineage phenotypes at week 4 of ATO initiated from single Ly- or My-HSC, shown as a percentage of ATOs with growth. Age groups are shown separately. Each dot represents an individual experiment. Error bar denotes +/- SD (n = 234 ATOs total from 6 independent experiments, ordinary two-way ANOVA). **g)** Frequencies of ATO lineage phenotypes at week 6 of ATO initiated from single Ly- and My-HSC, shown as a percentage of ATOs with growth. Age groups are shown separately. Each dot represents an individual experiment. Error bar denotes +/- SD (n = 275 ATOs total from 6 independent experiments, ordinary two-way ANOVA).

For all statistical analyses, only significant values are shown. A p value of <0.05 was deemed significant (p* ≤ 0.05, **p ≤ 0.01, ***p ≤ 0.001, ****p ≤ 0.0001).

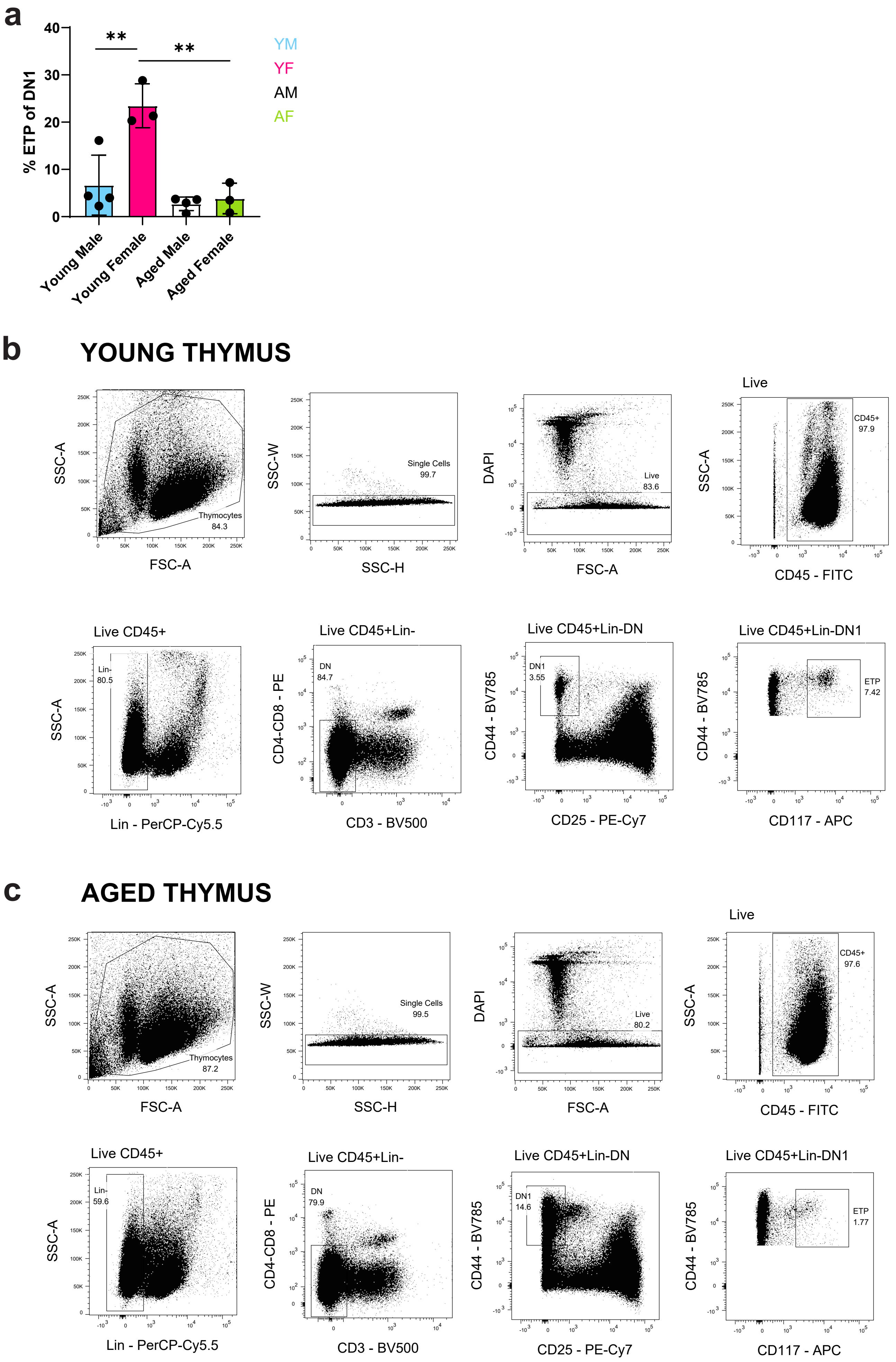

Supplemental Figure 7

Supplemental Figure 7. ETP frequencies and gating strategy from young and aged thymus.

a) Frequency of ETPs from young and aged male and female C57BL/6J mouse thymus, shown as a percentage of DN1 cells. Each dot represents an individual experiment with average of pooled data from 2-6 mice. Error bar denotes +/- SD (n = 54 mice, ordinary one-way ANOVA). **b)** Representative FACS plots of young thymus for ETP sort. Gates are shown as a percentage of the parent population. Data is concatenated from six independent experiments into one FACS plot. **c)** Representative FACS plots of aged thymus for ETP sort. Gates are shown as a percentage of the parent population. Data is concatenated from six independent experiments into one FACS plot.

YM = young male; YF = young female; AM = aged male; AF = aged female. For all statistical analyses, only significant values are shown. A p value of <0.05 was deemed significant ($p^* \leq 0.05$, $^{**}p \leq 0.01$, $^{***}p \leq 0.001$, $^{****}p \leq 0.0001$).

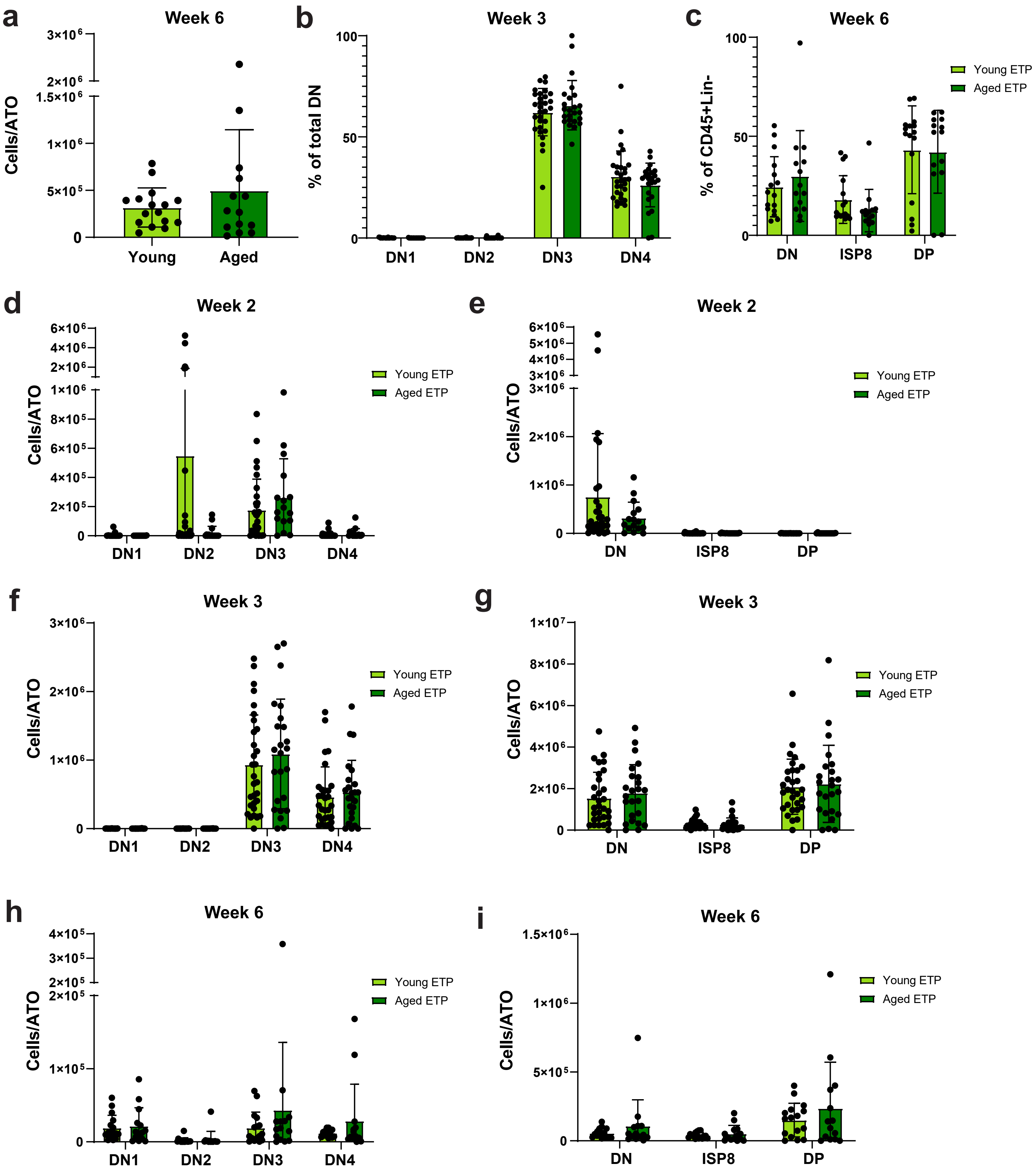

Supplemental Figure 8

Supplemental Figure 8. Differentiation kinetics and population cell numbers of single ETP ATOs.

a) Cell numbers at week 6 of ATO initiated from single ETP from young and aged C57BL/6J mice. Each dot represents 1 ATO. Error bar denotes +/- SD (n = 30 ATOs total from 4 independent experiments, Welch's t test). **b)** Frequencies of subsets of DN cells at week 3 of ATO initiated from single ETP from young and aged C57BL/6J mice, shown as a percentage of total DN cells. Each dot represents 1 ATO. Error bar denotes +/- SD (n = 54 ATOs total from 4 independent experiments, multiple unpaired t tests). **c)** Frequencies of subsets at week 6 of ATO initiated from single ETP from young and aged C57BL/6J mice. Frequencies of DN cells, ISP8 cells, and DP cells are shown as a percentage of total live CD45+Lin- cells. Each dot represents 1 ATO. Error bar denotes +/- SD (n = 30 ATOs total from 4 independent experiments, multiple unpaired t tests). **d)** Cell numbers of subsets of DN cells at week 2 of ATO initiated from single ETP from young and aged C57BL/6J mice, calculated using total cell numbers and frequency of live cells. Each dot represents 1 ATO. Error bar denotes +/- SD (n = 46 ATOs total from 4 independent experiments, multiple unpaired t tests). **e)** Cell numbers of subsets at week 2 of ATO initiated from single ETP from young and aged C57BL/6J mice, calculated using total cell numbers and frequency of live cells. Each dot represents 1 ATO. Error bar denotes +/- SD (n = 46 ATOs total from 4 independent experiments, multiple unpaired t tests). **f)** Cell numbers of subsets of DN cells at week 3 of ATO initiated from single ETP from young and aged C57BL/6J mice, calculated using total cell numbers and frequency of live cells. Each dot represents 1 ATO. Error bar denotes +/- SD (n = 54 total ATOs from 4 independent experiments, multiple unpaired t tests). **g)** Cell numbers of subsets at week 3 of ATO initiated from single ETP from young and aged C57BL/6J mice, calculated using total cell numbers and frequency of live cells. Each dot represents 1 ATO. Error bar denotes +/- SD (n = 54 total ATOs from 4 independent experiments, multiple unpaired t tests). **h)** Cell numbers of subsets of DN cells at week 6 of ATO initiated from single ETP from young and aged C57BL/6J mice, calculated using total cell numbers and frequency of live cells. Each dot represents 1 ATO. Error bar denotes +/- SD (n = 30 total ATOs from 4 independent experiments, multiple unpaired t tests). **i)** Cell numbers of subsets at week 6 of ATO initiated from single ETP from young and aged C57BL/6J mice, calculated using total cell numbers and frequency of live cells. Each dot represents 1 ATO. Error bar denotes +/- SD (n = 30 ATOs total from 4 independent experiments, multiple unpaired t tests).

For all statistical analyses, only significant values are shown. A p value of <0.05 was deemed significant ($p^* \leq 0.05$, $^{**}p \leq 0.01$, $^{***}p \leq 0.001$, $^{****}p \leq 0.0001$)

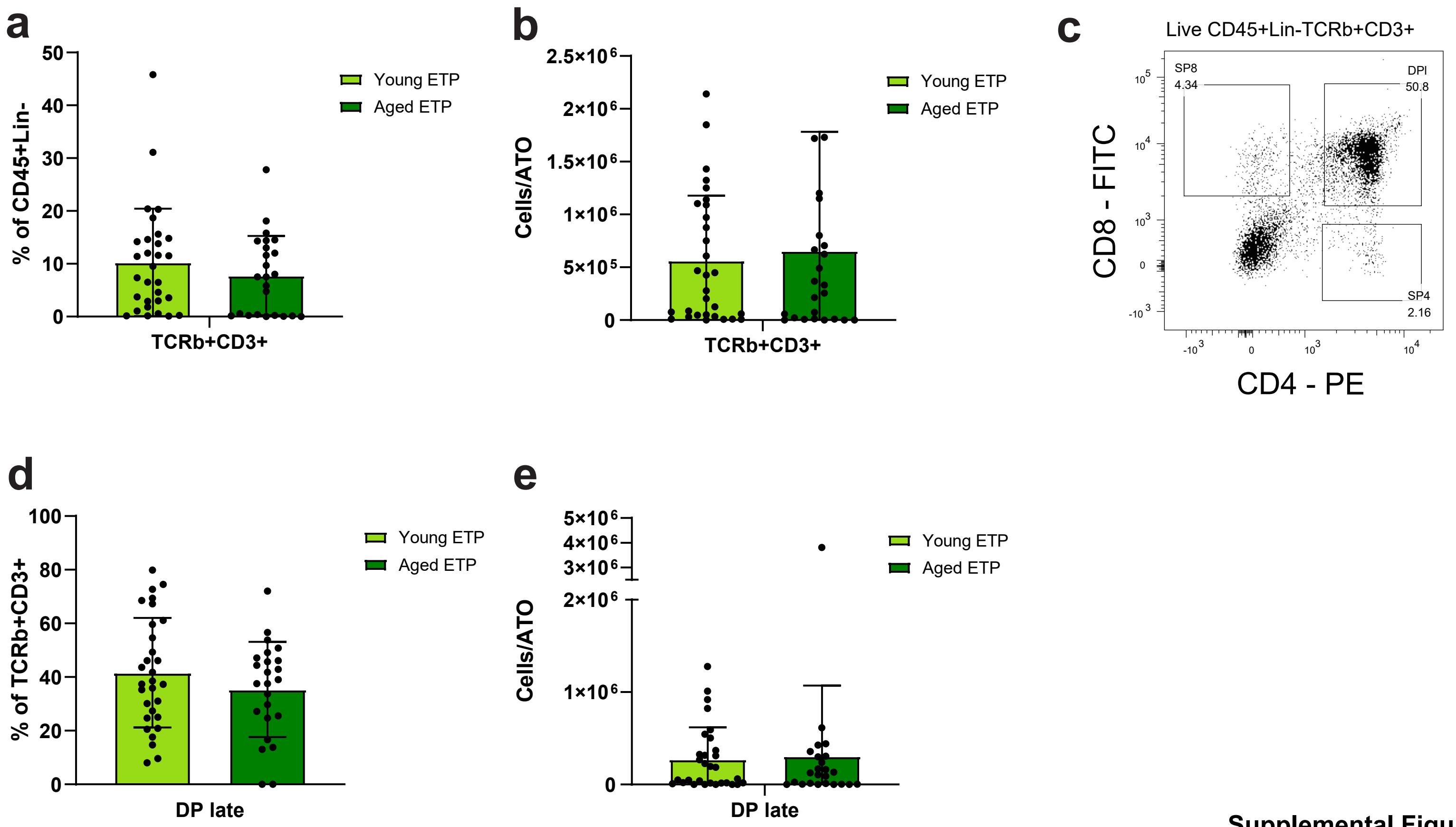

Supplemental Figure 9

Supplemental Figure 9. Differentiation kinetics and population cell numbers of mature cell subsets generated from single ETPs at week 3 of ATO.

a) Frequencies of TCRβ+CD3+ cells initiated from single ETP from young and aged C57BL/6J mice, shown as a percentage of total live CD45+Lin- cells. Each dot represents 1 ATO. Error bar denotes +/- SD (n = 54 ATOs total from 4 independent experiments, Welch's t test). **b)** Cell numbers of TCRβ+CD3+ cells initiated from single ETP from young and aged C57BL/6J mice, calculated using total cell numbers and frequency of live cells. Each dot represents 1 ATO. Error bar denotes +/- SD (n = 54 total ATOs from 4 independent experiments, Welch's t test). **c)** Representative FACS plot of one ATO initiated from single aged ETP with mature TCRβ+CD3+ subsets DP late (TCRβ+CD3+CD4+CD8+), SP4, and SP8. Frequencies are shown as a percentage of TCRβ+CD3+ cells. **d)** Frequencies of DP late cells (TCRβ+CD3+CD4+CD8+) initiated from single ETP from young and aged C57BL/6J mice, shown as a percentage of TCRβ+CD3+ cells. Each dot represents 1 ATO. Error bar denotes +/- SD (n = 54 ATOs total from 4 independent experiments, Welch's t test). **e)** Cell numbers of DP late cells (TCRβ+CD3+CD4+CD8+) initiated from single ETP from young and aged C57BL/6J mice, calculated using total cell numbers and frequency of live cells. Each dot represents 1 ATO. Error bar denotes +/- SD (n = 54 total ATOs from 4 independent experiments, Welch's t test).

For all statistical analyses, only significant values are shown. A p value of <0.05 was deemed significant (p* ≤ 0.05, **p ≤ 0.01, ***p ≤ 0.001, ****p ≤ 0.0001).

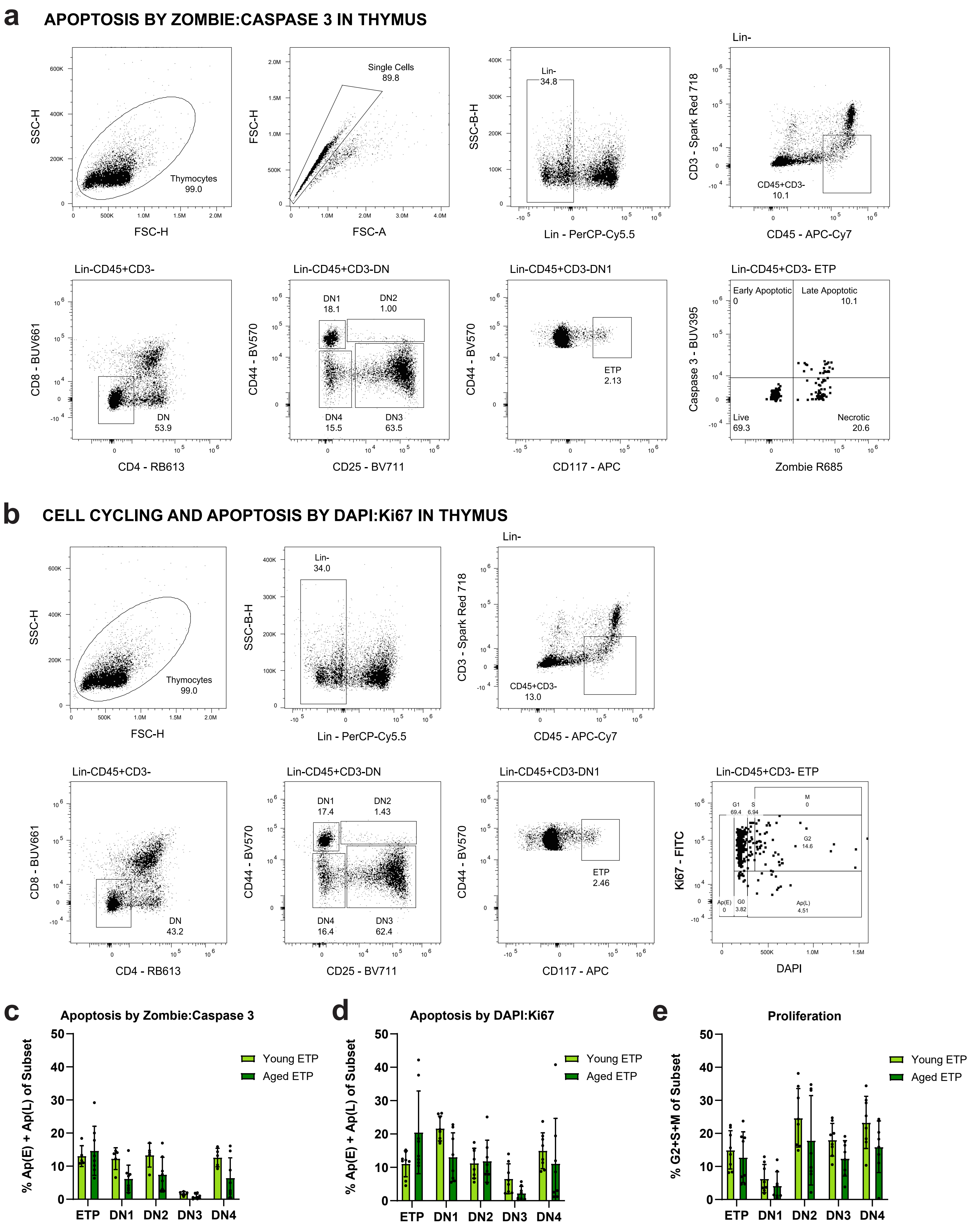

Supplemental Figure 10

Supplemental Figure 10. Apoptosis and cell cycling of early thymocytes from aged and young thymus.

a) Representative FACS plots of cell populations in Zombie:Caspase 3 apoptosis assay from young thymus. Gates are shown as a percentage of the parent population. **b)** Representative FACS plots of cell populations in DAPI:Ki67 cell cycling and apoptosis assay from young thymus. Gates are shown as a percentage of the parent population. **c)** Frequencies of early (Ap(E)) and late (Ap(L)) apoptotic cells from young and aged thymus using Zombie:Caspase 3 assay, shown as a percentage of the corresponding thymocyte subset. Error bar denotes +/- SD (n = 13 total mice from 3 independent experiments, multiple unpaired t tests). **d)** Frequencies of early (Ap(E)) and late (Ap(L)) apoptotic cells from young and aged thymus using DAPI:Ki67 assay, shown as a percentage of the corresponding thymocyte subset. Error bar denotes +/- SD (n = 16 total mice from 3 independent experiments, multiple unpaired t tests). **e)** Frequencies of proliferating cells in G2, S, and M phase from young and aged thymus, shown as a percentage of the corresponding thymocyte subset. Error bar denotes +/- SD (n = 16 total mice from 3 independent experiments, multiple unpaired t tests).

For all statistical analyses, only significant values are shown. A p value of <0.05 was deemed significant ($p^* \leq 0.05$, $^{**}p \leq 0.01$, $^{***}p \leq 0.001$, $^{****}p \leq 0.0001$).

Supplemental Table 1. Anti-mouse antibodies. Antibodies are listed based on relevant sort or developmental panel and include the name, fluorophore, source, catalog number, and clone.

HSC and LSK Sort

Flow cytometry antibody	*Source*	*Identifier*
Anti-mouse IL7R/CD127 (APC)	BioLegend	Cat#135011, Clone A7R34
Anti-mouse CD48 (APC-Cy7)	BioLegend	Cat#103432, Clone HM48-1
Anti-mouse Flk2/CD135 (PE)	BioLegend	Cat#135306, Clone A2F10
Anti-mouse Sca1 (PE-Cy7)	BioLegend	Cat#108114, Clone D7
Anti-mouse CD117 (BV605)	BioLegend	Cat#135122, Clone ACK2
Anti-mouse CD150 (BV711)	BioLegend	Cat#115941, Clone TC15-12F122
Anti-mouse CD3 (PerCP-Cy5.5)	BioLegend	Cat#100328, Clone 145-2C11
Anti-mouse B220 (PerCP-Cy5.5)	BioLegend	Cat#103230, Clone RA3-6B2
Anti-mouse Gr1 (PerCP-Cy5.5)	BioLegend	Cat#108428, Clone RB6-8C5
Anti-mouse NK1.1 (PerCP-Cy5.5)	BioLegend	Cat#108728, Clone PK136
Anti-mouse Ter119 (PerCP-Cy5.5)	BioLegend	Cat#116228, Clone TER119

ETP Sort

Flow cytometry antibody	*Source*	*Identifier*
Anti-mouse CD45 (FITC)	BioLegend	Cat#103107, Clone 30-F11
Anti-mouse CD25 (PE-Cy7)	BioLegend	Cat#102016, Clone PC61
Anti-mouse CD117 (APC)	BD Pharm.	Cat#553356, Clone 2B8
Anti-mouse CD117 (APC)	Invitrogen	Cat#17-1172-82, Clone ACK2
Anti-mouse CD3 (BV510)	BioLegend	Cat#100234, Clone 17A2
Anti-mouse CD44 (BV785)	BioLegend	Cat#103059, Clone IM7
Anti-mouse CD4 (PE)	BioLegend	Cat#130310, Clone H129.19
Anti-mouse CD4 (PE)	BioLegend	Cat#116006, Clone RM4-4
Anti-mouse CD8a (PE)	BioLegend	Cat#100708, Clone 53-6.7
Anti-mouse B220 (PerCP-Cy5.5)	BioLegend	Cat#103230, Clone RA3-6B2
Anti-mouse CD11b (PerCP-Cy5.5)	BioLegend	Cat#101228, Clone M1170
Anti-mouse Gr1 (PerCP-Cy5.5)	BioLegend	Cat#108428, Clone RB6-8C5
Anti-mouse Ter119 (PerCP-Cy5.5)	BioLegend	Cat#116228, Clone TER119

T cell development panels

Flow cytometry antibody	*Source*	*Identifier*
Anti-mouse CD8a (FITC)	BioLegend	Cat#100706, Clone 53-6.7
Anti-mouse CD8a (PE)	BioLegend	Cat#100708, Clone 53-6.7
Anti-mouse CD4 (PE)	BioLegend	Cat#130310, Clone H129.19
Anti-mouse CD4 (PE)	BioLegend	Cat#116006, Clone RM4-4
Anti-mouse CD4 (BV785)	BioLegend	Cat#100552, Clone RM4-5
Anti-mouse CD25 (PE-Cy7)	BioLegend	Cat#102016, Clone PC61
Anti-mouse CD3 (APC)	BioLegend	Cat#100312, Clone 145-2C11
Anti-mouse CD27 (APC-Cy7)	BioLegend	Cat#124226, Clone LG.3A10
Anti-mouse Thy1.2 (BV421)	BioLegend	Cat#140327, Clone 53-2.1
Anti-mouse Thy1.2 (AF700)	BioLegend	Cat#140324, Clone 52-2.1

Anti-mouse CD45 (APC-Cy7)	BioLegend	Cat#103116, Clone 30-F11
Anti-mouse CD45 (BV510)	BioLegend	Cat#103138, Clone 30-F11
Anti-mouse CD117 (BV605)	BioLegend	Cat#135122, Clone ACK2
Anti-mouse TCRβ (BV711)	BioLegend	Cat#109243, Clone H57-597
Anti-mouse TCRβ (BV510)	BioLegend	Cat#109234, Clone H57-597
Anti-mouse CD44 (BV785)	BioLegend	Cat#103059, Clone IM7
Anti-mouse CD19 (PerCP-Cy5.5)	BioLegend	Cat#152406, Clone ID3/CD19
Anti-mouse B220 (PerCP-Cy5.5)	BioLegend	Cat#103230, Clone RA3-6B2
Anti-mouse Gr1 (PerCP-Cy5.5)	BioLegend	Cat#108428, Clone RB6-8C5
Anti-mouse CD11c (PerCP-Cy5.5)	BioLegend	Cat#117328, Clone N418
Anti-mouse CD11b (PerCP-Cy5.5)	BioLegend	Cat#101228, Clone M1170
Anti-mouse NK1.1 (PerCP-Cy5.5)	BioLegend	Cat#108728, Clone PK136
Anti-mouse Ter119 (PerCP-Cy5.5)	BioLegend	Cat#116228, Clone TER119
Anti-mouse TCRγδ (PerCP-Cy5.5)	BioLegend	Cat#118118, Clone GL3
Anti-mouse TCRγδ (BV605)	BioLegend	Cat#118129, Clone GL3
Anti-mouse TCRγδ (BV421)	BioLegend	Cat#118120, Clone GL3
Anti-mouse NK1.1 (BV510)	BioLegend	Cat#108737, Clone PK136
Anti-mouse CD62L (BV605)	BioLegend	Cat#104438, Clone MEL-14

Myeloid panels

Flow cytometry antibody	*Source*	*Identifier*
Anti-mouse CD45 (FITC)	BioLegend	Cat#103107, Clone 30-F11
Anti-mouse CD45 (APC-Cy7)	BioLegend	Cat#103116, Clone 30-F11
Anti-mouse CD19 (AF700)	BioLegend	Cat#152414, Clone ID3/CD19
Anti-mouse CD19 (PE)	BioLegend	Cat#152408, Clone ID3/CD19
Anti-mouse B220 (PE)	BioLegend	Cat#103208, Clone RA3-6B2
Anti-mouse B220 (BV605)	BioLegend	Cat#103243, Clone RA3-6B2
Anti-mouse Gr1 (APC)	BioLegend	Cat#108412, Clone RB6-8L5
Anti-mouse Ly6G (PE-Cy7)	BioLegend	Cat#127618, Clone IA8
Anti-mouse CD8α (APC)	BD Pharm.	Cat#561093, Clone 53-6.7
Anti-mouse CD8α (PE)	BioLegend	Cat#100708, Clone 53-6.7
Anti-mouse CD8α (FITC)	BioLegend	Cat#100706, Clone 53-6.7
Anti-mouse CD11c (APC-Cy7)	BioLegend	Cat#117323, Clone N418
Anti-mouse I-A/I-E MHCII (BV421)	BioLegend	Cat#107631, M5/114.15.2
Anti-mouse CD11b (PE-Cy7)	BioLegend	Cat#101216, Clone M1/70
Anti-mouse CD11b (BV510)	BD Horizon	Cat#562950, Clone M1/70
Anti-mouse CD317 (BV605)	BioLegend	Cat#127025, Clone 927
Anti-mouse Ly6C (BV711)	BioLegend	Cat#128037, Clone HK1.4
Anti-mouse F4/80 (BV785)	BioLegend	Cat#123141, Clone BM8
Anti-mouse CD4 (PE)	BioLegend	Cat#130310, Clone H129.19
Anti-mouse CD4 (PE)	BioLegend	Cat#116006, Clone RM4-4
Anti-mouse CD4 (PerCP-Cy5.5)	BD Pharm.	Cat#550954, Clone RM4-5
Anti-mouse CD4 (PerCP-Cy5.5)	BioLegend	Cat#116012, Clone RM4-5
Anti-mouse CD4 (PerCP-Cy5.5)	BioLegend	Cat#100540, Clone RM4-4
Anti-mouse CD4 (BV711)	BioLegend	Cat#100550, Clone RM4-5
Anti-mouse CD3 (PE)	BioLegend	Cat#100308, Clone 145-2C11

Anti-mouse CD3 (PerCP-Cy5.5)	BioLegend	Cat#100328, Clone 145-2C11
Anti-mouse NK1.1 (PerCP-Cy5.5)	BioLegend	Cat#108728, Clone PK136
Anti-mouse NK1.1 (BV510)	BioLegend	Cat#108737, Clone PK136
Anti-mouse NK1.1 (BV421)	BioLegend	Cat#108732, Clone PK136
Anti-mouse Ter119 (PerCP-Cy5.5)	BioLegend	Cat#116228, Clone TER119

Apoptosis and cell cycling assays

Flow cytometry antibody	*Source*	*Identifier*
Anti-mouse CD45 (APC-Cy7)	BioLegend	Cat#103116, Clone 30-F11
Anti-mouse CD3 (Spark Red 718)	BioLegend	Cat#100282, Clone 17A2
Anti-mouse CD4 (RB613)	BD Horizon	Cat#571102, Clone GK1.5
Anti-mouse CD8α (BUV661)	Invitrogen	Cat#376-0081-82, Clone 53-6.7
Anti-mouse CD117 (APC)	BD Pharm.	Cat#553356, Clone 2B8
Anti-mouse CD25 (BV711)	BioLegend	Cat#102049, Clone PC61
Anti-mouse CD44 (BV570)	BioLegend	Cat#103037, Clone IM7
Anti-mouse/human Ki-67 (FITC)	BioLegend	Cat#151212, Clone 11F6
Anti-Active Caspase 3 (BUV395)	BD Pharm.	Cat#570187, C92-605.rMAb
Anti-mouse B220 (PerCP-Cy5.5)	BioLegend	Cat#103230, Clone RA3-6B2
Anti-mouse Gr1 (PerCP-Cy5.5)	BioLegend	Cat#108428, Clone RB6-8C5
Anti-mouse CD11b (PerCP-Cy5.5)	BioLegend	Cat#101228, Clone M1170
Anti-mouse NK1.1 (PerCP-Cy5.5)	BioLegend	Cat#108728, Clone PK136
Anti-mouse Ter119 (PerCP-Cy5.5)	BioLegend	Cat#116228, Clone TER119
Anti-mouse CD19 (PerCP-Cy5.5)	BioLegend	Cat#152406, Clone ID3/CD19
